# Supplementary material for: Structural Insights into De Novo Promoter Escape by Mycobacterium tuberculosis RNA Polymerase
Source: Nat Commun. 2025 Nov 13;16:9990. doi: 10.1038/s41467-025-64941-7 (PMC12615793; doi:10.1038/s41467-025-64941-7)
Supplement: Supplementary file 1 — Supplementary Information [file 41467_2025_64941_MOESM1_ESM.pdf]

Supplementary Information

Structural Insights into De Novo Promoter Escape by *Mycobacterium tuberculosis* RNA Polymerase

Joshua Brewer[1,2], Madeleine Delbeau[1], Winston Bates Zoullas[1,3], Seth A. Darst[2], Elizabeth A. Campbell[1*]

[1] Laboratory of Molecular Pathogenesis, The Rockefeller University, New York, NY 10065, USA.

[2] Laboratory of Molecular Biophysics, The Rockefeller University, New York, NY 10065, USA.

*Address correspondence to: campbee@rockefeller.edu

[3] Current address: Stanford University, Stanford, CA, 94305, USA

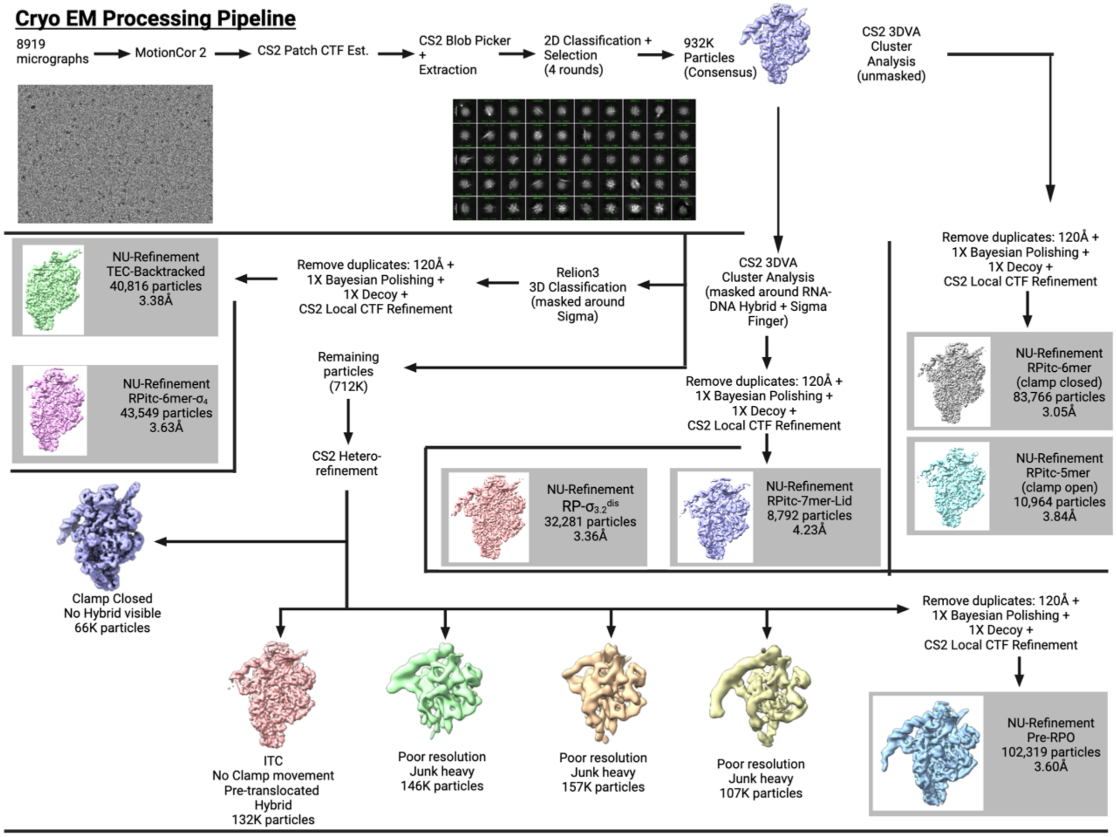

Supplementary Fig. 1 | Cryo-EM processing pipeline for promoter escape structures.

Created in BioRender. Campbell, E. (2025) https://BioRender.com/tknav7g

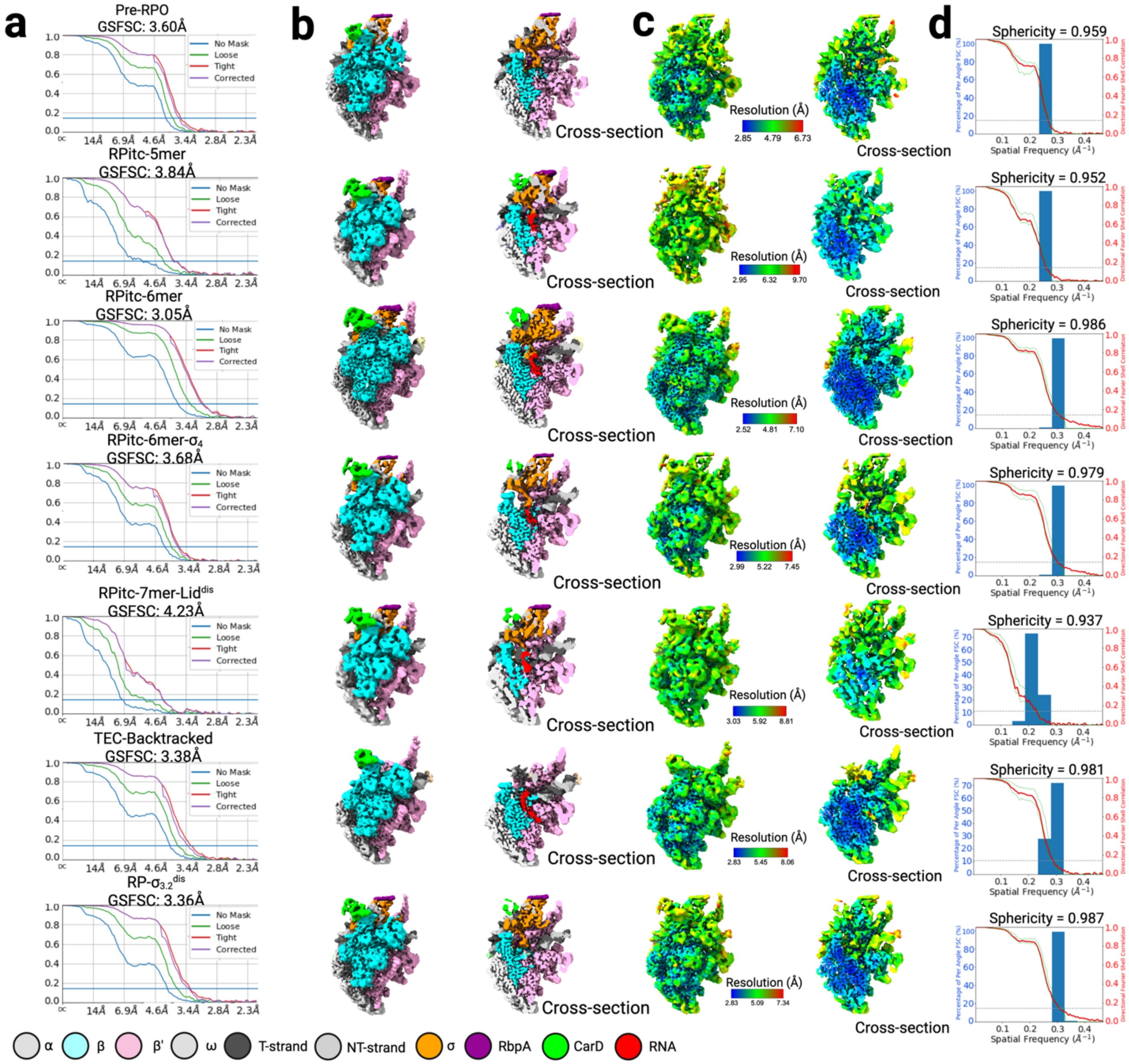

Supplementary Fig. 2 | CryoEM for promoter escape structures

a. Gold standard FSC calculations for cryo-EM maps.

b. Cryo-EM maps and cross-sections are colored according to the key (bottom of figure). RNAP is colored pink for the β′ subunit and cyan for the β subunit, with ω and the α subunits in light grey. σ is shown in orange, CarD in green, and RbpA in purple. The DNA template strand (T-strand) is dark grey, and the non-template strand (NT-strand) is light grey, with the –10 element in magenta and the –35 element in yellow. The nascent RNA is colored red.

c. Cryo-EM maps are colored according to local resolution (with the associated legend for heat maps).

d. 3DFSC and sphericity of cryo-EM maps.

Created in BioRender. Campbell, E. (2025) https://BioRender.com/tknav7g

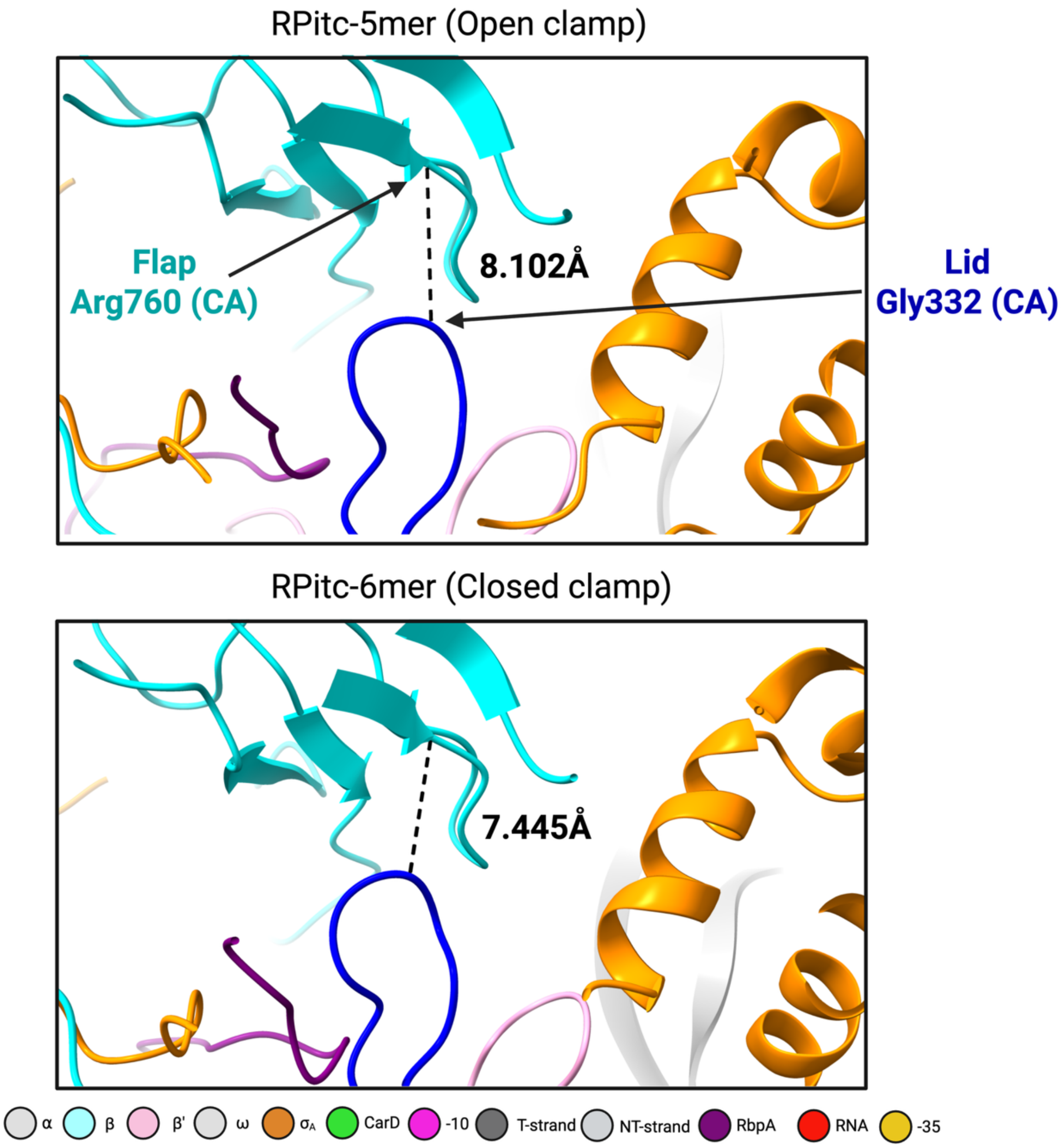

Supplementary Fig. 3 | Clamp opening in early promoter escape has minimal effect on the distance between the β flap and β' lid.

Comparison of the open clamp RPitc-5mer (top panel) and the closed clamp RPitc-6mer (bottom panel), with Arg760 of the β flap and Gly332 of the β' lid remaining similarly spaced in both promoter complex intermediates, suggesting alternative mechanisms responsible for successful σ finger escape. RNAP is colored pink for the β′ subunit and cyan for the β subunit, with ω and the α subunits in light grey. σ is shown in orange, the lid in blue, and RbpA in purple. Created in BioRender. Campbell, E. (2025) https://BioRender.com/tknav7g

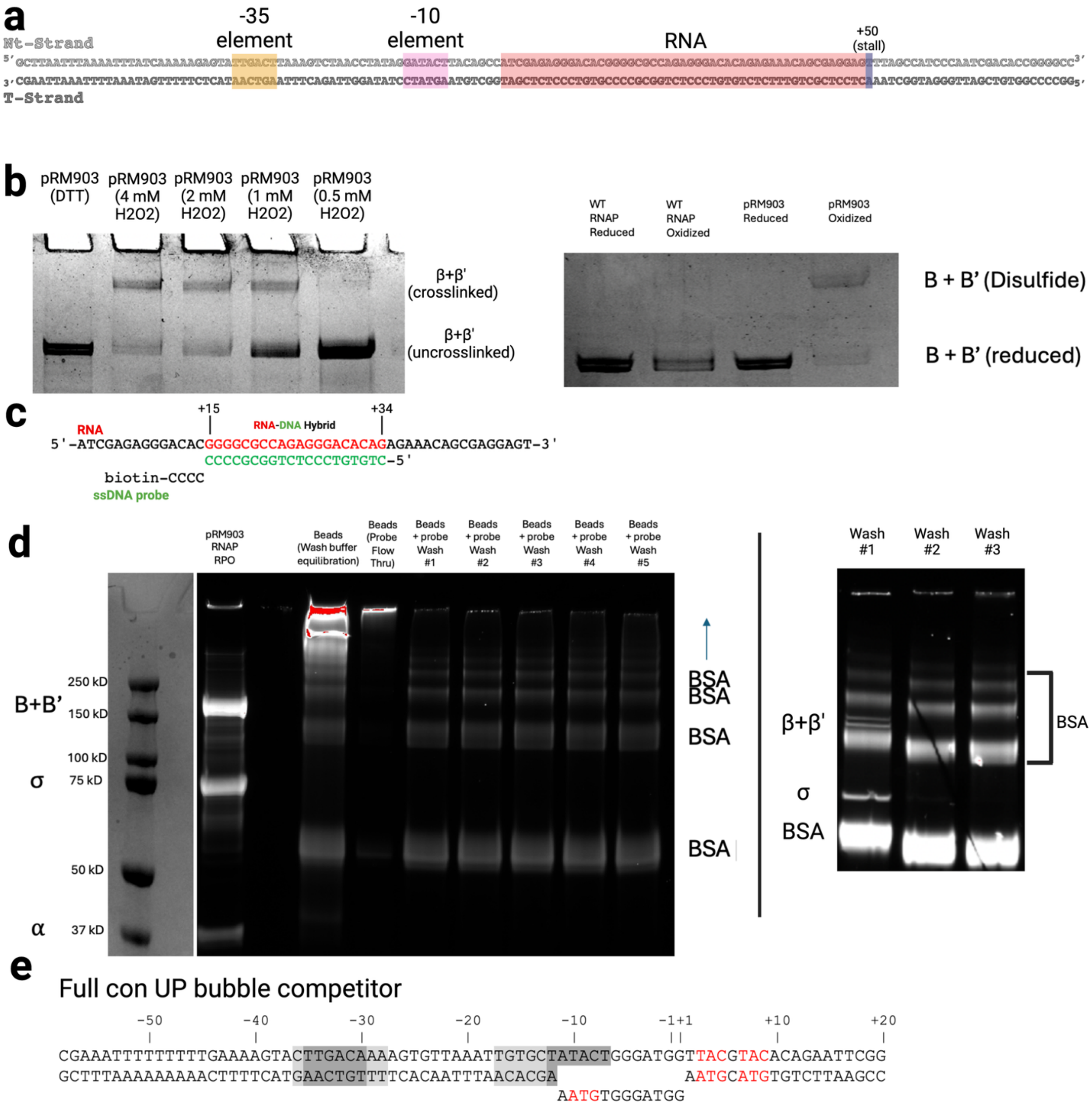

Supplementary Fig. 4 | Experimental controls for σ-retention pulldown assay.

a. dsDNA scaffold designed for stalling transcription at +50 after initiating via T7A1 promoter.

b. pRM903 (free core RNAP) shows dose-dependent crosslinking sensitivity to DTT and H2O2. Based on these results, a final concentration of 2 mM H_2O_2 was used in subsequent assays (shown on the left). WT RNAP does not show crosslinking behavior when directly compared to pRM903 at 5mM H2O2 (shown on the right).

c. Design of ssDNA biotinylated probes hybridized to RNA generated via de novo transcription initiation from dsDNA scaffold described in Extended Data Fig. 4a

d. SDS page results comparing elements of transcription reaction employed in σ-retention pulldown (1st labelled lane after ladder) with a sample containing only magnetic beads and wash buffer without transcription reaction elements (2nd labelled lane), a sample containing flow-through from the probe-labelling of the magnetic beads with no wash buffer present (3rd labelled lane) and samples containing only magnetic beads, wash buffer, and ssDNA probe subject to iterative rounds of washing (4th-8th labelled lanes) [all shown on the left]. These results directly implicate the BSA present in the wash buffer in the production of additional noted bands. Aggregated BSA bands are distinct from RNAP and σ and are disregarded in the final σ-retention pulldown analysis. Stringent washes of streptavidin-coated magnetic beads remove unbound RNAP and σ by wash #2-3 while continually producing additional BSA bands (shown on the right).

e. Full con UP bubble competitor DNA used in pulldown transcription reactions to prevent multi-round transcription.

Created in BioRender. Campbell, E. (2025) https://BioRender.com/tknav7g

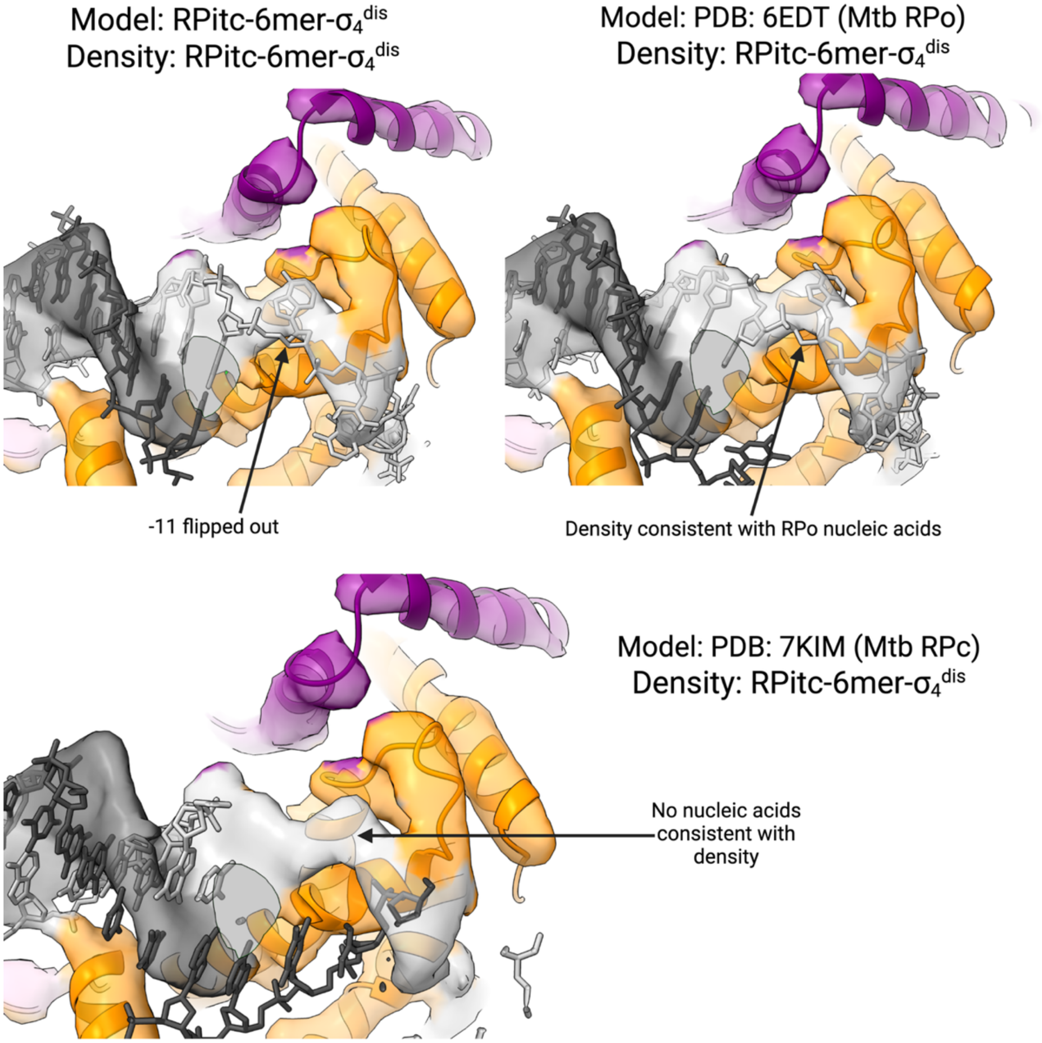

Supplementary Fig. 5 | -10 promoter element appears to be intact in RPitc-6mer-σ_4^{dis}

Cryo-EM map from RPitc-6mer-σ_4^{dis} (pictured in all three images above) was used to build models based on preexisting known structures of Mtb RPo. All structural models shown above have been 3D-aligned based on σ. The final model for RPitc-6mer-σ_4^{dis} is shown in the top left. RPitc-6mer-σ_4^{dis} cryo-EM map predicts the 'flipped-out' state of the -11 base of the core -10 promoter element in the known RPo structure of Mtb RNAP (shown top right). The aligned RPc structure (shown bottom left) displays positioning of nucleic acids inconsistent with the cryo-EM map from RPitc-6mer-σ_4^{dis}. These results indicate that the -10 element is intact in RPitc-6mer-σ_4^{dis} while the -35 core promoter element is disrupted. σ is shown in orange, and RbpA in purple. The DNA template strand (T-strand) is dark grey, and the non-template strand (NT-strand) is light grey.

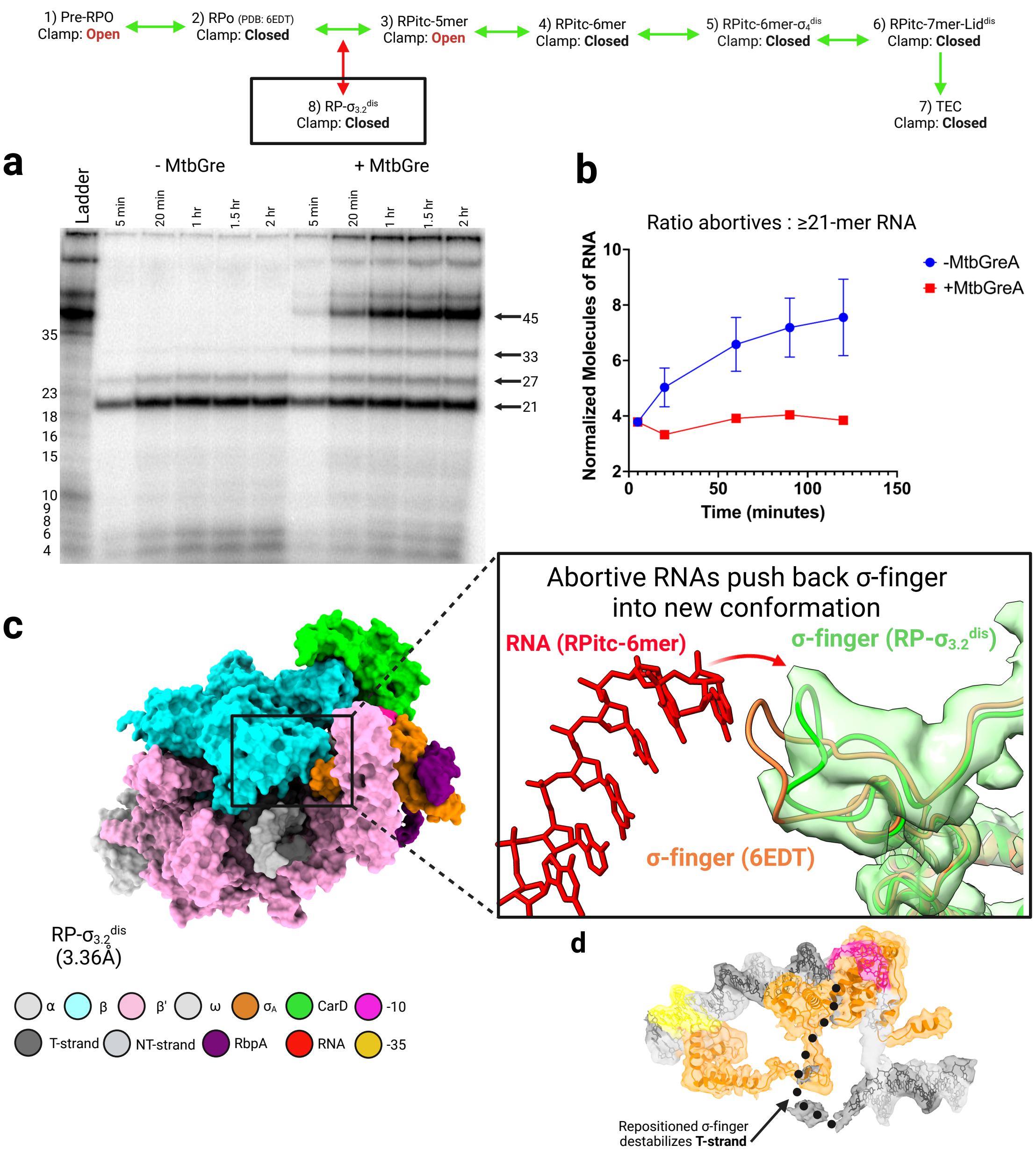

Supplementary Fig. 6 | MtbGreA stimulates promoter escape but is unlikely to target off-pathway RP-$\sigma_{3.2}^{dis}$ complex

a. Radioactive transcription gels illustrate how the MtbGre factor stimulates promoter escape. Source data are provided as a Source Data file.

b. Quantification of transcription gels illustrates how the MtbGre factor stimulates promoter escape. Ratios calculated between =>21-mer RNA (corresponding to escaped ECs) and abortive RNA (3-9nt). Comparison of ratios observed in MtbGre+ and MtbGre- conditions is plotted. Error bars denote standard error. All data are presented as mean values +/- SD. All data are presented as mean values +/- SD. Sample size (n) = 2 independent experiments. Source data are provided as a Source Data file.

c. Cryo-EM structure of RP-$\sigma_{3.2}^{dis}$ complex. The color key in the structure is shown below. The panel highlights the reposition of the σ finger in RP-$\sigma_{3.2}^{dis,}$ consistent with the direction of extending RNA (shown here from RPitc-6mer). The position of the σ-finger in RPo (PDB: 6EDT) is shown for comparison. RNAP is colored pink for the β′ subunit and cyan for the β subunit, with ω and the α subunits in light grey. σ is shown in orange. The DNA template strand (T-strand) is dark grey, and the non-template strand (NT-strand) is light grey, with the –10 element in magenta and the –35 element in yellow. Panel on the right shows the resting σ-finger in orange (6EDT) and from RPitc-6mer in green.

d. Repositioning of σ-finger appears to destabilize T-strand DNA in RP-$\sigma_{3.2}^{dis}$. The carved cryoEM map is shown. σ is shown in orange, CarD in green, and RbpA in purple. The DNA template strand (T-strand) is dark grey, and the non-template strand (NT-strand) is light grey, with the –10 element in magenta and the –35 element in yellow. The nascent RNA is colored red.

Created in BioRender. Campbell, E. (2025) https://BioRender.com/tknav7g

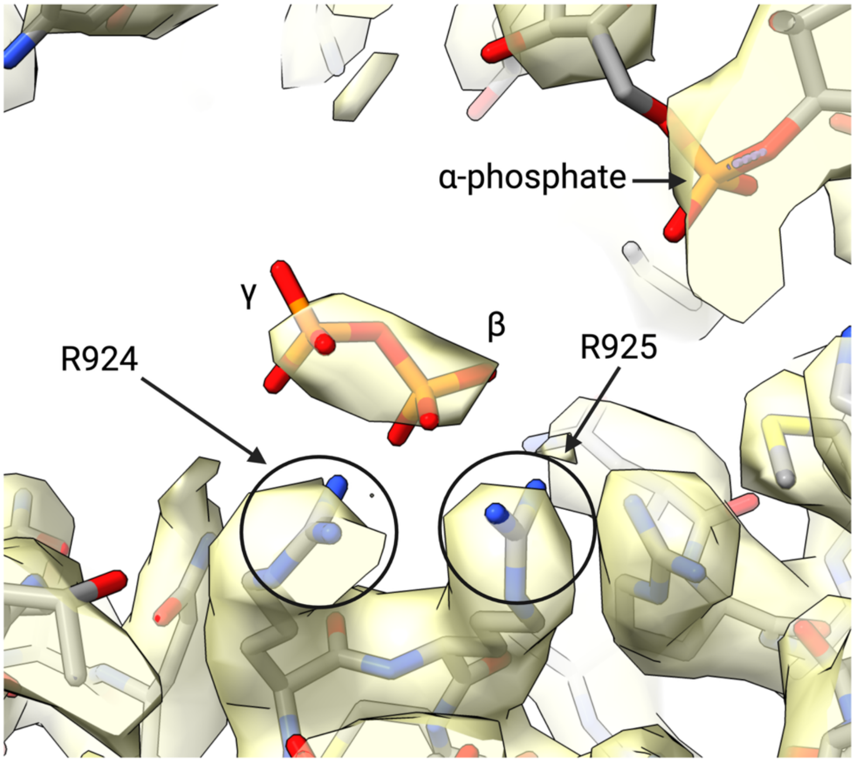

Supplementary Fig. 7 | Structural analysis of *Mtb*'s pyrophosphate binding pocket.

Cryo-EM map (RPitc-6mer) shows pyrophosphate (ppi) bound in the pocket with positively charged guanidine groups (circled ends of side chains) of sequential arginine residues (βR924, βR925) closely contacting the negatively charged pyrophosphate.

Created in BioRender. Campbell, E. (2025) https://BioRender.com/tknav7g

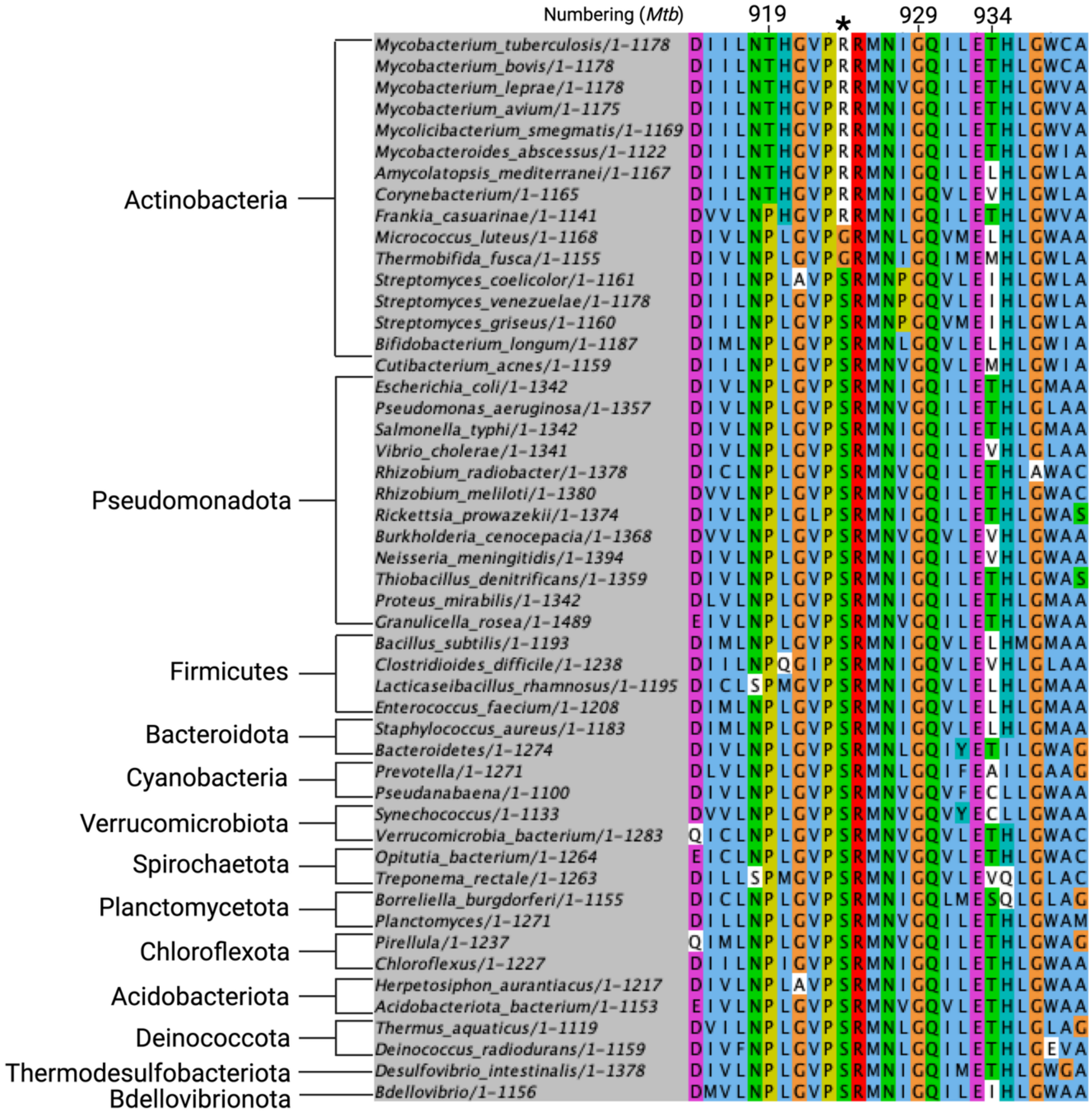

Supplementary Fig. 8 | Phylogenetic analysis of *Mtb*'s pyrophosphate binding pocket.

Multiple sequences of alignment of RpoB (coding for RNAP subunit β) within various species across major bacterial phyla (Uniprot). Sequential arginine residues contributing to the pyrophosphate binding pocket are noted in the table above (*). The 'RR' motif appears to be restricted to Actinobacteria, and this motif is chiefly present in Mycobacteria-proximal clades.

Created in BioRender. Campbell, E. (2025) https://BioRender.com/tknav7g

Supplementary Note 1

B-factor modification in Pymol with Python Code

Purpose - Replace the B-factors of a given protein in Pymol with their standard deviation from the structure's mean B-factor, creating a more standardized metric of variation across structures. Used to show variation in B-factors between the CarD transcription factors in the RPtic-5mer (with open clamp) and the RPtic-6mer (with closed clamp).

Procedure - Open a new session of Pymol. Type in the terminal "set pdb_retain_ids, 1" so that the script maintains the IDs of individual particles (see [PyMOL wiki - pdb_retain_ids]). Import your structure into pyMOL. Select and name whatever part of your molecule you hope to modify, creating one "reference object" (this can be the whole molecule) and one "target object" (the named selection).

Go into files and find "run script", and navigate to "ScaleBs.py" (the code listed below, which should be saved beforehand and converted into a .py file). Selecting this will change your directory to the proper working directory, allowing for use of the command. To actually use the command, type in the terminal "ScaleBs [obj_1_name], [ref_obj_name]". This should have the effect of changing the color of atoms of the selected object based on their deviation from the mean. The code only does this for the alpha carbons in the structure, so the command "AlphaToAll" can be used to extend these B-factors to the rest of the residue to emphasize the visual effect (see [PyMOL wiki - AlphaToAll]).

To standardize fully, enter the command "spectrum b, minimum=-4, maximum=4" - the value 4 here is what was found as the extremal deviation for our structures, but can be modified as long as it's kept consistent between the structures being compared. This command can also take the selection as an argument if you want to just recolor the selected area by spectrum, and can also be used to modify the colors in the spectrum (see [pymol.org - Command: spectrum]).

Design - ScaleBs uses the numPy python library to enable easy manipulation calculations of B-factors. It takes inspiration from the loadBfacts command (see [PyMOL wiki - loadBfacts]) to actually interface with pyMOL, replacing the B-factors. The program is written to download both the selected object and the reference object as new PDB files, read them, and filter through to get the desired data. It sorts for only alpha carbons and accounts for common inconsistencies in the formatting of a PDB file, namely how the space between the B-factor column and the last position column disappears if the B-factor goes past 100, and how the space between chain letter and the residue number disappears if the residue number passes 1000.

The code then adds the index of each alpha carbon in the selected object data to a list, every B-factor to a different list, and every B-factor of the reference to a different list. It converts these two lists of B-factors into numPy arrays, then computing the average of the reference list and the standard deviation. It uses these to create a new array of scaled B-factors, computed in the following way:

bf_scaled = (np.round(((bf_obj - avg_ref)/std_ref)*100))/100

In other words, the average of the reference is subtracted from each element in the list of the selected object's B-factors, then divided by the standard deviation in the reference, and then rounded to the second decimal place. This produces a new list which encodes the B-factor's standard deviation from the reference's mean. This list is converted into a dictionary, attaching the proper indices to the corresponding b values, and reads the B-factors back into PyMOL with the "cmd.alter()" command (see [pymol.org - Command : alter]).
