# Supplementary material for: Structural Insights into De Novo Promoter Escape by Mycobacterium tuberculosis RNA Polymerase
Source: Nat Commun. 2025 Nov 13;16:9990. doi: 10.1038/s41467-025-64941-7 (PMC12615793; doi:10.1038/s41467-025-64941-7)
Supplement: Supplementary file 2 — Description of Additional Supplementary Files [file 41467_2025_64941_MOESM2_ESM.docx]

File Name: Supplementary_Data_File_1.txt
Description: Primary protein sequences for RpoB (RNAP β subunit) from analyzed species (Supplementary Fig. 8) located via UniProt and collated into a FASTA-format data file.

File Name: Supplementary_Note_1.docx
Description: Details on purpose and design of B-factor analysis code

File Name: Supplementary_Software_1.py

Description: Python code for B-factor analysis
