# Supplementary material for: Structural Insights into De Novo Promoter Escape by Mycobacterium tuberculosis RNA Polymerase
Source: Nat Commun. 2025 Nov 13;16:9990. doi: 10.1038/s41467-025-64941-7 (PMC12615793; doi:10.1038/s41467-025-64941-7)
Supplement: Supplementary file 3 — Supplementary Dataset 1 [file 41467_2025_64941_MOESM3_ESM.pdf]

File Name: Supplementary_Data_File_1
Description: Primary protein sequences for RpoB (RNAP β subunit) from analyzed species (Supplementary Fig. 8) located via UniProt and collated into a FASTA-format data file.

File Name: Supplementary_Software_1.py

Description: Python code for B-factor analysis

Supplementary_Software_1.py
Description: Python code for B-factor analysis

```
from pymol import cmd, stored, math
import numpy as np
import os

# defining function
def ScaleBs (obj,ref_obj, min=-4, max=4):
    # save objects
    cmd.save("obj.pdb",obj,-1)
    cmd.save("ref_obj.pdb",ref_obj,-1)
    obj_path = "obj.pdb"
    ref_path = "ref_obj.pdb"

    # read the files
    with open(obj_path) as f:

        data_obj = f.readlines()

    with open(ref_path) as f:
        data_ref = f.readlines()



    b_factors_obj = []
    b_factors_ref = []


    # sort through selected and grab B-factors
    for line in data_obj:
        if "CA" in line:
            CA_line = line
            n = CA_line.split()
            if len(n[5]) < 4:
                if len(n[9]) > 4:
                    slicedB = n[9][4:]
                    b_factors_obj.append(float(slicedB))
                else:
                    unslicedB = n[10]
                    b_factors_obj.append(float(unslicedB))
            else:
                if len(n[8]) > 5:
                    slicedB = n[8][4:]
                    b_factors_obj.append(float(slicedB))
                else:
                    unslicedB = n[9]
                    b_factors_obj.append(float(unslicedB))
```

```
# sort through ref pdb and grab B-factors
for line in data_ref:
    if "CA" in line:
        CA_line = line
        n = CA_line.split()
        if len(n[5]) < 4:
            if len(n[9]) > 4:
                slicedB = n[9][4:]
                b_factors_ref.append(float(slicedB))
            else:
                unslicedB = n[10]
                b_factors_ref.append(float(unslicedB))
        else:
            if len(n[8]) > 5:
                slicedB = n[8][4:]
                b_factors_ref.append(float(slicedB))
            else:
                unslicedB = n[9]
                b_factors_ref.append(float(unslicedB))

bf_obj = np.array(b_factors_obj)
bf_ref = np.array(b_factors_ref)

# calculate scaled B-factors from ref average and stdev
avg_ref = np.mean(bf_ref)
std_ref = np.std(bf_ref)
bf_scaled = (np.round(((bf_obj - avg_ref)/std_ref)*100))/100
bf_sc = bf_scaled.tolist()


indx = []

# record indices of selected obj
for line in data_obj:
    if "CA" in line:
        CA_line = line
        n = CA_line.split()
        indx.append(int(n[1]))


b_dict = {}

# make dictionary
for i,val in enumerate(indx):
    b_dict[val] = bf_sc[i]

#write in B-factors
for k,v in b_dict.items():
    cmd.alter("%s and id %s and n. CA"%(obj,k), "b=%s"%v)
```

```
    # color objects (rescaling of min and max may be necessary)
    cmd.show_as("cartoon",obj)
    cmd.spectrum("b","rainbow", "%s and n. CA " %obj)
    cmd.ramp_new("scaling", obj, [min, max], "rainbow")
    cmd.recolor()

    # remove created pdbs
    os.remove("obj.pdb")
    os.remove("ref_obj.pdb")

# implement function
cmd.extend("ScaleBs", ScaleBs);
```

Primary protein sequences for RpoB (RNAP β subunit) from analyzed species (Supplementary Fig. 8) located via UniProt and collated into a FASTA-format data file.

```
>sp|Q9KWU7|RPOB_THEAQ DNA-directed RNA polymerase subunit beta
OS=Thermus aquaticus OX=271 GN=rpoB PE=1 SV=1
MEIKRFGRIREVIPLPPLTEIQVESYKKALQADVPPEKRENVGIQAAFKETFPIEEGDKG
KGGLVLDFLEYRIGDPPFSQDECREKDLTYQAPLYARLQLIHKDTGLIKEDEVFLGHLPL
MTEDGSFIINGADRVIVSQIHRSPGVYFTPDPARPGRYIASIIPLPKRGPWIDLEVEASG
VVTMKVNKRKFPLVLLLRVLGYDQETLVRELSAYGDLVQGLLDEAVLAMRPEEAMVRLFT
LLRPGDPPKKDKALAYLFGLLADPKRYDLGEAGRYKAEEKLGVGLSGRTLVRFEDGEFKD
EVFLPTLRYLFALTAGVPGHEVDDIDHLGNRRIRTVGELMADQFRVGLARLARGVRERMV
MGSPDTLTPAKLVNSRPLEAALREFFSRSQLSQFKDETNPLSSLRHKRRISALGPGGLTR
ERAGFDVRDVHRTHYGRICPVETPEGANIGLITSLAAYARVDALGFIRTPYRRVKNGVVT
EEVVYMTASEEDRYTIAQANTPLEGDRIATDRVVARRRGEPVIVAPEEVEFMDVSPKQVF
SLNTNLIPFLEHDDANRALMGSNMQTQAVPLIRAQAPVVMTGLEERVVRDSLAALYAEED
GEVVKVDGTRIAVRYEDGRLVEHPLRRYARSNQGTAFDQRPRVRVGQRVKKGDLLADGPA
SEEGFLALGQNVLVAIMPFDGYNFEDAIVISEELLKRDFYTSIHIERYEIEARDTKLGPE
RITRDIPHLSEAALRDLDEEGIVRIGAEVKPGDILVGRTSFKGEQEPSPEERLLRSIFGE
KARDVKDTSLRVPPGEGGIVVGRLRLRRGDPGVELKPGVREVVRVFVAQKRKLQVGDKLA
NRHGNKGVVAKILPVEDMPHLPDGTPVDVILNPLGVPSRMNLGQILETHLGLAGYFLGQR
YISPVFDGATEPEIKELLAEAFNLYFGKRQGEGFGVDKREKEVLARAEKLGLVSPGKSPE
EQLKELFDLGKVVLYDGRTGEPFEGPIVVGQMFIMKLYHMVEDKMHARSTGPYSLITQQP
LGGKAQFGGQRFGEMEVWALEAYGAAHTLQEMLTIKSDDIEGRNAAYQAIIKGEDVPEPS
VPESFRVLVKELQALALDVQTLDEKDNPVDIFEGLASKR
>sp|Q9RVV9|RPOB_DEIRA DNA-directed RNA polymerase subunit beta
OS=Deinococcus radiodurans (strain ATCC 13939 / DSM 20539 / JCM
16871 / CCUG 27074 / LMG 4051 / NBRC 15346 / NCIMB 9279 / VKM B-1422 /
R1) OX=243230 GN=rpoB PE=3 SV=2
MTLSKTPPRIERFGDITEVIPLPNLTEVQVNSFKAFLQDDKAPDQREDVGLQSAFREVFP
IDESEKGRSTGMVLDFIEYRLGEPEYSPEECREKDLTYEAPLYVKLELIHKDTGVIKGFK
PDSPPESWVFLGNLPLMTFDGSFIINGADRVVISQIHRSPGVYFTSSYKGIKKQYTAAII
PMPKRGPWIELEFAGDVLEMKVNKRKFPVSLLLRVLGMDDASIRALFTEFTPEVEPGEDK
SAGMGADEALLRLFTVLRPGDPPKRDKAIQYLFGLLADPRRYDLGEPGRFKMNTKLGVQR
QERTLLKFEDGKFSDAGLVDTIRYLMALQQGLETVPMVDEDGVVTDVPVAEDDIDHLGNR
RVRTVGELLADQLRVGMGRMARGVRERMLLGNPDAATPTKLVNNRPIVAAMREFFGRSQL
SQFKDQTNPLSDLRHKRRISALGPGGLTRERAGFDVRDVHRTHYGRICPIETPEGANIGL
ISSLSSYAKVNDLGFIMAPYRKVEDGKVTNQVEYMTADIEDRYTIAQANSPLNEDNTFAD
ERVLARRKGDPLLYTPDEVDYMDVSPKQIVSINTSLIPFLEHDDANRALMGSNMQSQAVP
LVRADSPAVGTGVERRVVTDSGTSVVSDVNGRVSYVDARAIQVTLSEDHRELNMNAGNVR
TFELIRFTRSNQGTNLDQHPIVSVGDEVKVGQVIADGPASERGRLALGQNITIAIMPFDG
FNFEDAICINEDLVRQDFYTSVHIEKDEIEARDTKLGPEKITRDIPGLSEAALRDLDEDG
IVRVGAEVKPGDILVGKTSFKGESEPTPEERLLRSIFGEKAREVKDTSLRVQSGQGGIVV
KTVRFRRGDEGVDLKPGVREMVRVYVAQKRQLQVGDKVANRHGNKGVVSKIVRPEDMPYL
EDGTPVDIVFNPLGVPSRMNLGQILETHLGEVARLTGQKFETPVFDSVTEATIKEMLEVA
AAERLQARKDDGFELDKREQEVLDRAGKLGVIDAPGDDYEKGQMQLARTGKSILYDGRTG
EPISGPVVVGIMYVMKLYHMVEDKLHARSTGPYSLITQQPLGGKAQFGGQRFGEMEVWAL
EAYGAAHVLQEMLTIKSDDIDGRDAAYQSIVKGEEVSGSTIPESFKVLVKELHSLGLDVE
```

VLDHGDKAVDIFEGMMPKR
>tr|A0A239ME78|A0A239ME78_9BACT DNA-directed RNA polymerase subunit beta OS=Granulicella rosea OX=474952 GN=rpoB PE=3 SV=1
MSEMRAIRSRLDFSKIPTSIQIPNLIEVQRRSYERFLQMDKLPQEREDNGLQSVFTSVFP
ITDFRNVSELEFVDFSIGNWECKCGYLKGLNHLRTACSHCGHMVITDPFHPGDVLCNFCG
TYNKNTPDFCTKCGDPVGLQLKYDQAECEERGMTYSAPLKVTIRLKIYDKDPETGVKSLR
DMKEQEVFFGDIPLMSQNGTFIVNGTERVIVSQLHRSPGVFFETANNRTYFLGKIIPYRG
SWVEFEYDQKNTLYVRIDRKRKFLGTIFLRALGLKSDEDILKTFYTVDTIKVAEGKLSWV
VAEDGKPTNLLGTRPSSSVKVAGDEIAVGGRKVSPSALKGLRSHKIEAVEVETSEFDGAM
IAADVVDMSTGELLYEANQELTADKLHKIIQSGVTSFEVFFPERDDVGNIITNTLRRDSV
RKPEEALIEIYRKLRPGDPPTLDTATALFEGMFFDPRKYDFSRVGRLKFNIKLYENQDPS
GLDMRTLTPEDFYGTIRYLLKLRKNIGVVDDIDHLGNRRVRAVGELMENQFRIGLVRMER
AIKEKMSVYQEMSTAMPHDLINAKPVMAAIREFFGSSQLSQFMDQTNPLSEITHKRRLSA
LGPGGLSRERAGFEVRDVHPTHYGRICPIETPEGPNIGLISSLSCFARINEYGFIESPYR
RVKDGRVLDYVAVANAGESGLRQGDYLEISESVTLNAKLKAEGKRTMDLEPFSFYLSAWE
EDRHTIAQANIQLDETLHIVQDIVDARRQGNFVLVNKAEVDYVDVSPKQLVSVAASLVPF
LEHDDANRALMGANMQRQSVPLLVAEAPFVGTGMEGVTARDSGAVILAKRNGIIDSVDSE
RIIVRVEGEHHPTQLSREVGSDIYQLTKFKRSNQNTCINQKPIVRKGDRVLKGQVIADGP
CTEQGELGLGRNVLVAFMPWRGYNFEDAILISEKLVREDYYTSIHIEEFEIEARDTKLGP
EEITRDIPNVSEHALRDLDESGIIRIGAKIGHNDILVGKVTPKGETQLTPEEKLLRAIFG
EKAGDVRDASLTCPPGIEGTVVDVRIFSRKGQEKDERAKQIEQEMVEKLERNLADEIRIL
TDERLKRLEAILGGKEVLADLHDERTNKKLLNKGEVLDRDMIELISTRNLKRIRYADKDP
RVNEQIDEIEEMTSRQIDVLRKITNEKIGKMQKGDELSPGVIKMVKVYIAMKRRLSVGDK
MAGRHGNKGVVARILPEEDMPYLPDGTPVEIVLNPLGVPSRMNVGQILETHLGWAAHTLG
AQIAELASKMSEANEVRELFKARFAGTAALNQLLNLDDEQTLRVAAGMKRGIWFGTAVFD
GAHESEIKALLKSAGLPSSGKTQLFDGMLGEPFEQPATVGYIYMLKLSHLVDDKIHARSI
GPYSLITQQPLGGKAQFGGQRFGEMEVWALEAYGAAYILQELLTAKSDDVFGRTKIYEAI
VKGEAAIEPGVPESFNVLIRELQSLCLDVELVKVADQKKVALPQIAAAD
>tr|A0A2E6NGM9|A0A2E6NGM9_UNCAI DNA-directed RNA polymerase subunit beta OS=Acidobacteriota bacterium OX=1978231 GN=rpoB PE=3 SV=1
MDEIIQLFYSVSSLHIRQGTLHWQVNENLLGRSAGATITVPGTEVSVKAGKKITKTLLQA
LVEAGIEEVEVSDAELEGAYSATDVVDPSTGEVILEANEEMTPRVVAMAQERSVTNLEIF
FPESDEIGSVLSQSLRKDSIRTHEEALIEIYRRMRPGDPPTLESSRTLFENMFFNAQKYD
FSRVGRLKLNTKLGVDTSLDEKVLRPVDFFQVIKYLLKLKRNPANVDDIDHLGNRRVRSV
GELLENQFRIGLVRMERAIKEKMSVYQEMATAMPHDLINAKPVMAAIREFFGSSQLSQFM
DQTNPLSEITHKRRLSALGPGGLSRERAGFEVRDVHPTHYGRICPIETPEGPNIGLISSL
SCYARINEFGFIESPYRRVRDGRVNESVVIVNAGSTKFKIGDVVEVEDVLEENSAAKSRK
KRGADYEPHSFYLSAWEEDRYVIAQANSGVDESGSLIDERVNARQAGDFVLAPREKIEYI
DVSPKQLVSVAASLIPFLENDDANRALMGSNMQRQAVPLLRARAPFVGTGMEXITARDSG
SVVSAKRSGTVDYVDSKRXVVRVDGXGDTSXDMGADXYNLTKFKRSNQNTCIXQKXIAXV
GQHXXKGQVLADGPCTELGELALGRNVLVAFMPWRGYNFEDAVLVSERMVKEDYYTSIHV
EEFDVEARDTKLGPEEITRDIPNVSEGFLNDLDESGIIRIGAPVKPGDILAGKVTPKGET
QLTPEEKLLRAIFGEKAGDVRDASLTCPPGIEGIVVGVKIFSRKGIEKDERAKAIEAEDL
EGLEKNLQDEIRILHDEVKKRIIHMLPGQVLDADLFDEFGRERLLSQGVALTPELLLDLP
YEAIARMKINFDDVRLEEDLRALKDRTGNQVEVTRQLFEERREKIRRGDELPPGVIKLVK
VYVAMKRKLSVGDKMAGRHGNKGVIARILPEEDMPYLPDGTPVEIVLNPLGVPSRMNVGQ
VLETHLGWAAHALGLYFSTPVFDGATESEIKGWLDQAGLPKTGKTELFDGMTGQAFEQDV
TVGYIYMLKLSHLVDDKIHARSIGPYSXVTQQPLGGKAQFGGQRFGEMEVWALEAYGAAX
ILQELLTAKSDDVIGRAKIYEAIVKDDASFTPGLPESFNVLVRELQALCLDIELVKKKPX
VIEVNPEPVLEEV

>sp|A9B6J3|RPOB_HERA2 DNA-directed RNA polymerase subunit beta OS=Herpetosiphon aurantiacus (strain ATCC 23779 / DSM 785 / 114-95) OX=316274 GN=rpoB PE=3 SV=1
MPQVQSTVILPPLITSSIFRDDQGRAMIASRRSFARITDAIELPKLIETQIDSFRWFQRE
GLRELFDEINPIDDFTGKNLELSFSDYEFGEPRYNEFECRERDMTYAAPLRVRVRLKVKT
TGEIKESDIFLGDFPLMTNNGTFVINGAERVVVSQLIRSPGVYFKEEKEPTSGRSLHSAK
LIPNRGAWLEFETNKRDVLSVKVDRKRKLPVTILIRAVLGLFGDKPLDQCGLNEDVIALF
ANVDVNRDHQYIASTLDKDPSTNAKEAIMELYKRLRPGDPATVDNARSLLETLLFNARRY
DLGKVGRYKLNRNLWEKSRLFEGQEIPSLTMRVLSKADLFKIVERLIDLNNGIGNPDDID
HLGNRRVRTVGELIQTQFRVGLLRMERVIKERMSLQEPEAATPNGLINIRPVVAAMREFF
GGSQLSQFMDQTNPLAELTHKRRLSALGPGGLSRDRAGFEVRDVHHSHYGRICPVETPEG
PNIGLIGTMSTFARVNEMGFLETPYRRVYREVDNAPLWLERGMTTRDVRHLSTGELIARA
GARVDAELAKIIAIGVLNGQLLREDIVNPATGDLIAEAGSEIDRALATKIADLPLRAIKI
HPVVTNETDYLPADEEDKFIIAQANALLDERFRFIDPTVSCRHAEDFVQAPIASVDYMDV
SPKQVVSVSTALIPFLEHDDANRALMGSNMQRQAVPLLRPDAPIIGTGMEHKAARDSGQV
VVARADAVVLSSNSERILVRENDGTERTYPLLKFLRSNQDTCINQRPSVFIGDKIRAGEV
IADSSSTQNGELALGQNILVAYMPWEGGNFEDAILVSERLVREDIFTSIHIEKYEVEARD
TKLGPEEITRDIPNVGQDSLKNLDERGIIYVGADVQPNDILVGKITPKGETDLTAEERLL
RAIFGEKAREVKDSSLRVPNGVKGKVIDVKVFTRDETTEMPVGVNQTVRVMLCQKRKISP
GDKMAGRHGNKGVVSRVLPIEDMPFLPDGTPVDIVLNPLAVPSRMNIGQILETHLGWAAS
RLGFRIATPVFDGARDEDIIKALAESGLPSDAKIDLYDGRTGEKFDNPVTVGYKYMLKLA
HLVEDKIHARSTGPYSLVTQQPLGGKAQFGGQRFGEMEVWALEAYGAAYILQEMLTVKSD
DVVGRVKTYEAIVKGDPINEAGVPESFKVLIKELQSLGLSVDVLTADERPVELTDSEADD
LISLDGINLSGMEKGEL
>tr|A0A2A2RVD4|A0A2A2RVD4_9BACT DNA-directed RNA polymerase subunit beta OS=Opitutia bacterium Tous-C10FEB OX=1982318 GN=rpoB PE=3 SV=1
MADRTHTERINFGKLREVIQPPNLIELQISSYLEYLQKDTPEKQRKPYGLEAVFKEVFPI
HSYDERLTLEYVSYTIGEPKSSEIECLREGVTYAVPLYVKLRLREEDFIKDEEIFMGDIP
MVTERGSFIINGAERVVVSQLHRSPGICFEVATHPNGKLLHSFRIIPDRGTWLEVQFDNN
DLLYVYLDRRRRRRKFLITTLLRSIGFSNDLDILNLFYTIQDLKVSKALDLDNVSTLALV
EDVIDAQKGVVLARAFEPLTKAIVRTFEKHDIKSMRVIDTTADEGAIIRALKKDPTRNEE
EALKEVYKKLRPGEPPTPANAKALLKRLFFDPKRYDLGRVGRYKINQKLDLKMDLENRIL
DSADIVAATKYLVRLKKSDGIVDDIDHLGSRRVRTVGELLANQCRVGLSRTERLVRERMT
LYDQSVDSITPQKLINPKALTTVIRDFFARSQLSQFMDQINPLAELTHKRRLSALGPGGL
NRERAGFEVRDVHPSHYGRICPIETPEGPNIGLINSLSTYARVNEFGFIESPYRVVEKGR
VSDKVVYLTADQEEGKTIAQANAEVDSKGHYVGKVTARHSGNFLEVSAEEVDLMDVSPKQ
VVSVAAGLIPFLEHDDANRALMGSNMQRQGVPLLQTEAPFVGTGLEARLASDSKTVVIAE
EAGLVASVDAKQIIVTKDGELPRHPKHDPKNHVFVYELRKFMRSNAGTCFSQRPIVKKGQ
KVKKGECIADGPSTDHGELALGRNVLVGFMPWNGYNFEDAILISEKVLKEDIFTSIHVQE
FEVIARDTKLGPEEITRDIPNIGEEALKHLDHNGVIRIGAEVKPGDLLVGKITPKSETEL
APEEKLLRAIFGEKAADVKDTSLIVPSGVNGIIMDVKVSTSRLDAERDRLSPSDRRRQVK
QIQEDYKTQMDKLREQLTEALSNILLGEKIPLDVINGQTGEIIIPANRKITKTLLRKLAA
VAKHIEIDPSPVRIKIMEIIASFQSKFDELEGDRERKIATIETGDENGPGVIKTVKVYIA
TKQKLEVGDKMAGRHGNKGVVAKIVPEEDMPFLPDGTPIEICLNPLGVPSRMNVGQVLET
HLGWACKKLGIKVATPVFDGIPESKVRGYLKEANLPMSGKSPLHDGRTGEKLDQEVVVGY
IYMMKLNHLVSHKIHARAVGPYSLVTQQPLGGKAQYGGQRFGEMEVWALEAYGAAHTLQE
LLTVKSDDVNGRTKIYESLVKGDNTLSAGTPESFNVLVKEMQALGLDIKLAKRTALGEAL
GGSR
>sp|Q2JX64|RPOB_SYNJA DNA-directed RNA polymerase subunit beta OS=Synechococcus sp. (strain JA-3-3Ab) OX=321327 GN=rpoB PE=3 SV=1

MTQLAVPSPAAPTLPDLVEIQRESFLWFLREGFEEELLSFSPIVDYTGKLELHFLPEYRP
GDPSKGYKINKPRYDPEEAKRRDATYQAQIRVPTRLINKETGEIKDMDVFIGELPLMTDR
GTFIINGAERVIVNQIVRSPGVYYKSELDKNGRRTYSASLIPNRGAWLKFETDKNGLVWV
RIDKTRKLSAAVLLKALGLSDSEIYDSLRHPEFFQKTMEKEGHYSEEEALMELYRKLRPG
EPPTVSGGQQLLESRFFDPKRYDLGRVGRYKLNKKLNLNVAENVRVLTVTDILAVIDYLI
NLEYDIGHVDDIDHLGNRRVRSVGELLQNQVRVGLNRLERIIRERMTVSESENLTPASLV
NPKPLVAAIKEFFGSSQLSQFMDQTNPLAELTHKRRLSALGPGGLSRERAGFAVRDIHPS
HYGRICPIETPEGPNAGLIGSLATHARVNQYGFIESPYYRVENGVVRKDLGMVYLTADEE
DEYRVAPGDVPVDAEGRITADLVPVRYRQEFTTAHPSEVHYVQVSPVQLISVATSLIPFL
EHDDANRALMGANMQRQAVPLLKPDRPYVGTGLEAQAARDSGMVVVSRTSGVVTYVSADE
IVVRPDDGGDPIVYRLQKYQRSNQDTCLNQRPLVYAGDRVVPGQVLADGPATEGGELALG
QNVLVAYMPWEGYNYEDAILISERLVYDDVFTSVHIEKYEIEARQTKLGPEEITREIPNV
GEDALRNLDENGIVRIGAWVEAGDILVGKVTPKGESDQPPEERLLRAIFGEKARDVRDNS
LRVPNGERGRVVDVRIFTREQGDELPPGANMVVRVYIALKRKIQVGDKIAGRHGNKGIIS
RILPIEDMPYLADGTPVDVVLNPLGVPSRMNVGQVYECLLGWAAEHLGVRFKLMPFDEMH
GLEASRLTVEAKLREAREKTGKDWIFNPEGKYCGKIQVFDGRTGEPFDQPVTVGRAYMLK
LVHLVDDKIHARSTGPYSLVTQQPLGGKAQQGGQRFGEMEVWALEAFGAAYILQELLTVK
SDDMVGRNEALNAIVKGKPIPRPGTPESFKVLVRELQSLCLDVSVHKVEVDSDGQTRDVE
VDLMADVSSRHTPSRPTYESVTSEDLSPAAGGTFTLARRSREEDEDREEEDDF
>tr|A0A2W6YW45|A0A2W6YW45_9CYAN DNA-directed RNA polymerase subunit beta OS=Pseudanabaena sp OX=1153 GN=rpoB PE=3 SV=1
MNKPTLVTPAFVLPDLVEIQRESFRWFLEEGLIEELESFSPITDYTGKMELHFIAKDYKL
KRPKYSVDDSKRRDATYAVQMYVPTRLINKETGEIKEQEVFIGDLPLMTDRGTFIINGAE
RVIVNQIVRSPGVYYKQEIDKNGRRTYNASLIPNRGAWLKFETDKNDLVWVRIDKTRKLS
AQVLLKAIGLSDAEILDALTHREYFQKTIDKEGQFDEDEALKELYRKLRPGEPPTESGGR
ELLRSRFFDPKRYDLGKVGRYKINKKLRLNTPDTMRVLTEKDILTAINYLINLKFDIGEI
DDIDHLGNRRVRSVGELLQNQVRVGLNRLERIIRERMTVSDVDSLTPASLVNPKPLVAAI
KEFFGSSQLSQFMDQTNPLAELTHKRRLSALGPGGLTRERAGFAVRDIHPSHYGRICPIE
TPEGPNAGLIGSLATHARVNQYGFIETPYYAVENGKVLKNQEPIYMTADEEDEFRVAPGD
VATNDEGYIFSEIVPIRYRQEWGTATPEEIDYVAVSPVQIISVATSLIPFLEHDDANRAL
MGSNMQRQAVPLLRPERPLVGTGLEAQAARDSGMVIVSRVTGEVSYVSADEIRVKADDTG
LESIYRLQKYQRSNQDTCLNQRPLVWVGDTVVAGQVLADGSATEGGEIALGQNILVAYMP
WEGYNYEDAILINERLVIDDVYTSIHVEKYEIEARQTKLGPEEITREIPNVGEESLRNLD
EQGIIRIGAWVSSGEILVGKVTPKGESDQPPEEKLLRAIFGEKARDVRDNSLRVPNGEKG
RVVDVRVFTREQGDELPPGANMVVRVYVAQKRKIQVGDKMAGRHGNKGIISRILPKEDMP
FLPDGTPLDIVLNPLGVPSRMNVGQVFECLLGWAAENLNARFKIVPFDEMYGEEASRELV
HGQLEHARTHTGKDWVFNDEFPGKLTVYDGRTGEPFDQPVTVGKAYMLKLVHLVDDKIHA
RSTGPYSLVTQQPLGGKAQQGGQRFGEMEVWALEAFGAAYILQELLTVKSDDMTGRNEAL
NAIVKGHAIPRPGTPESFKVLVRELQSLCLDVSVHKLSDDGSNQDTEVDLMVDTGSRRTP
NRPTYESVYRGDINFDEDDD
>tr|A0A9D8MFT7|A0A9D8MFT7_9BACT DNA-directed RNA polymerase subunit beta OS=Prevotella sp OX=59823 GN=rpoB PE=3 SV=1
MASKKKVDTRINFASVQNPMPYPDFLDVQLKSFRDFLQLDTPPEERKNDGLYKVFAENFP
ITDTRNNFVLEFLDYFIDPPRYSIDECLERGLTYSVPLKAKMKLYCTDPDHEDFGTVTSD
VFLGTIPYMTSNGTFIINGAERVVVSQLHRSPGVFFGQGVHANGTVLYSARIIPFKGSWI
EFATDINNVMYAYIDRKKKLPVTTLLRAIGFEQDKDILQIFDLAEEVKVNKKNMKEAIGR
KLAARVLKSWNEDFVDEDTGEVVSIERNEVIMERETELTADNIAEILDSGASSVLLHKDA
EMASKFSIIFNTLAKDPSNSEKEAVLYIYRQLRNADPADDNSAKEVFMNLFFSDKRYDLG
EVGRYRINKKLGLDTDMNVRVLTKDDIIEIIKYLIQLVNSNATVDDIDHLSNRRVRTVGE
QLSNQFSIGLARMSRTIRERMNVRDNEVFTPTDLINAKTISSVINSFFGTNPLSQFMDQT

NPLAEVTHKRRLSALGPGGLSRERAGFEVRDVHYTHYGRLCPIESPEGPNIGLISSLCVY
ATINELGFIETPYRKVNGGVVDLDNENVVYLTAEEEAEHIIGQGNAPLKKNGSFIRTYVK
CRQDADFPVVAPSQVDLMDVSPQQIASVSAGLIPFLEHDDGHRALMGCNMMRQAVPLLHN
DAPIVGTGLEKQVCEDSRTMIVAEGDGVIEYVDATTIRILYDRTEDEEFVSFEPALKEYR
IPKFRRTNQNMTIDLRPLCKKGQRVSKGDILTEGYATENGELALGRNLLVAYMPWKGYNY
EDAIVLSERLVRDDVLTSVHVDEYSLDVRETKRGMEEFTADIPNVSEEATKDLDENGVIR
VGARVEPGDILIGKISPKGESDPSPEEKLLRAIFGDKAGDVKDSSLKANPSLSGVIIDKK
LFTRAVKTRESKKQDKVLLAKLEEEYEAKNNDLRDILIEKLMKLTRGKTSQGVKDYTGAE
IVTKGSKFSAATLRNLEYDGIQSSGWTGDEHKDMLIQKLIMNYLRKYKLLDAELKRRKFA
ISIGDELPSGILQMAKVYVAKKRKIGVGDKLAGRHGNKGIVSKVVRMEDMPFLEDGRPVD
LVLNPLGVPSRMNLGQIFEAILGAAGRKLGVKFATPIFDGAKLDDLSQWTDKAGLPRLCS
TRLYDGETGEQFDQPATVGVTYFLKLGHMVEDKMHARSIGPYSLITQQPLGGKAQFGGQR
FGEMEVWALEAFGASHVLQEILTIKSDDTVGRSKAYEAIVKGDPMPAAGIPESLNVLLHE
LRGLGLSVKLD
>tr|A0A7C2KLD4|A0A7C2KLD4_9CHLR DNA-directed RNA polymerase subunit
beta OS=Chloroflexus sp OX=1904827 GN=rpoB PE=3 SV=1
MPPLIESVVLQPLIAPIDPGADGRRSRRIERRSFARIKDAIDLPLLIETQLKSFEWFKRE
GLRELFDEISPITDFTGKNLELHFRDYTFGEPRYDEFECRERDLTYAAPLRVRVELRILT
TGEIKESEIFLGDFPIMTDNGTFVYNGAERVVVSQLIRSPGVYFKDEKEPTSGRSLHTAK
LIPNRGAWLEFETNKRDVISVKVDRKRKIPVTILLRAITAWIAEENGNGRWVPDNELDKF
GHNDQIIELFRHVDTVPEHPYIHATLDKDPSRNAKEALLELYKRLRPGDPPTLENARSLI
ESLLFSPRRYDLAKVGRYKLNKNLWERDVRRDGAKAPDLSVRVLLPRDIFRIVEQLILLN
NGHGRPDDIDHLGNRRVRTVGELIQQQFRVGLLRLERVVKERMSLQDPASATPNGLINIR
PVVAAMREFFGGSQLSQFMDQTNPLAELTNKRRLSALGPGGLSRDRAGFEVRDVHHSHYG
RICPVETPEGPNIGLIGTMSTFARVNEMGFLETPYRKVYNSVDNVQVWKEKGILLRDVRD
LRTGDLIAAKGTRVNDEIARQITIGLLRGQILREDIVDPDTDELIAEAGTEINRALAERI
VELPIKHIKIRPVVSQEVDYLSADEEDRFVIVQANAPLDQHNRFLDTIVSCRFGEDFVSE
RVERVDYMDVSPKQVVSVSTSLIPFLEHDDANRALMGSNMQRQAVPLLRPDAPIVGTGME
YRAARDSGQVIVARRDGVVVSTTSERIVIEEDDGNQTEYRLRKFMRSNQDTCINQRPAVV
RGQRVKAGDVIADSSSTDQGELALGQNVLVAYMPWEGGNFEDAILVSERLVREDIFTSIH
IEKYEVEARDTKLGPEEITRDIPNVGQESLRNLDERGIIYIGAEVQPNDILVGKITPKGE
TDLTAEERLLRAIFGEKAREVKDSSLRVPNGVRGKVIDVKVFSRSEGAELPVGVNQTVRV
LLCQKRKISAGDKMAGRHGNKGVVSRVLPIEDMPFLPDGRPVDIILNPIGVPSRMNIGQI
LETHLGWAAARLGFRVATPVFDGAHEDQIKDLLVQAGLPADGKVTLYDGRTGERFDNPVT
VGYAYMLKLAHLVEDKIHARSTGPYSLVTQQPLGGKAQFGGQRFGEMEVWALEAYGAAYT
LQEMLTVKSDDVVGRVKTYEAIVKGEPIQEAGVPESFKVLIKELQSLGLSVEVLSADEKP
VELSDDLDSDIGALEGINLSGMERGEF
>tr|A0A0P7YYW4|A0A0P7YYW4_9BACT DNA-directed RNA polymerase subunit
beta OS=Bacteroidetes bacterium HLUCCA01 OX=1666909 GN=rpoB PE=3 SV=1
MSNSYQTLPFTERISFARTKNVLEYPDFLDVQLESFRKFSQWNIQPEERADEGLQEIFLE
HFPIQDTREQHILEFLYYSIDTPRYTIKECQERGLTFSVPLKAKLRLSAVDQTDEASETI
EQEVFLGDLPWMTERGTFIINGAERVIVSQLHRSPGVFFGQSLHPNGTQLYSARIIPFKG
SWIEFTTDIRDVLWAYIDRKKKLPVTTLLRALGFSTDYDILRMFDLSEETEIKTKKAFVE
LIGKRLATDVAQELIEEVVDEETGEVKEAKRRMVILARDHEIAEEDYDILKEAGVDKVLT
LKISSEDAERSVILNTLRKDATYNEETALAEVYRQIRTGEMPDTETARQVLERLFFSDKK
YDLGEVGRYRLNKRLHLDEPLETTVLTINDIVAIIKELIRLKNLKSQVDDIDHLSNRRVR
TVGEQLAQQFALGLARMARTIRERMNSRDAEQFTPQDLVNARTISSVINTFFGTNQLSQF
MDQTNPLAELTHKRRMSALGPGGLTRERAGFEVRDVHYTHYGRLCPIETPEGPNIGLISS
LCVHAKVNEFGFIETPYRRVESGKVTDKVEYLAAEQEDETIIAQANAELTEKSTFKNEFI
FSRFRDSNVGLARPEQVEFMDVSTNQITSVAAALIPFIEHDDANRALMGSNMQRQGVPLL

RPERPVVGTGMEYRAARDSRALVVAEGAGEVVYVSATEIRIRYERDENEALAYFDEGVVT
YKLRKFERTNQDTTSNQIPIVKVGDKVVANQPLADGCATNRGELALGRNLLVAFMPWRGY
NFEDAIVISERVVSEDLYTSIHIQEFEQQVRDTKRGEEELTREIPNVSEEATRNLDENGI
IRVGAKVKPGDIIVGKITPKGETDPTPEEKLLRAIFGDKAGDVKDASLKTPPGVQGVVID
TRLFSRKRDEQYTRKEEKELVDREQQDFDRKIAKLNASWSERMYKLLKDKTSPGILDRAG
NEVIPKSEKYRKQTFEELDPMELRVNQAWCQDDNTVNIVVKLFENYRNVRRDYETELKRR
KYSIQVGDELPPGIVQKAKVYVAKKRKLQVGDKMAGRHGNKGVVAKIVPVEDMPFLEDGT
PVDIVLNPLGVPSRMNLGQIYETILGWAGKKLGVNFATPIFDGAKYDDVQYWLDQAGLPV
DGRTYLYDGRSGERFAQKTTVGYMYILKLNHLVQDKMHARSIGPYSLITQQPLGGKAQFG
GQRLGEMEVWALYAYGAASILKEMLTVKSDDVKGRSKVYEAIVKGENLPEGNTPESFNVL
LRELQGLGLEVNIE
>tr|A0A923BF49|A0A923BF49_9BACT DNA-directed RNA polymerase subunit beta OS=Pirellula sp OX=117 GN=rpoB PE=3 SV=1
MAVTAQRRLVTQNIRSFGSGRESFDLPDLTQIQTESYERFLQYNGGSPAKRKSEGLEAVL
QEIFPIESYDKTIKLEYLRYELGKPRYSMDECRQLRLTYGRPFRIWLQLNKETPVVEEVY
LGDLPIMLGGGEFIINGAERVVVSQLHRSPGIDFVSEMDPGERRMYSCRVIPERGSWIEL
NTTKKDSVTVRIDQSGKFSAMTLLRAMSPELGSDAEILRRFYPVTKQKVVDGRSAGKLEG
KVAVEDVVYPGESERAGEIIIESGQRITKNVAEIICTSGVKQVDVMDPPKTPHLLNALTD
DNTSSHEEALLRIYQRLRPGNPPQLEKARALFSEKFFDANRYRLGRVGRFRMNRKLNLNV
AETEMVLRPEDLLAAINYLLLLVAGENEAYVDDIDHLGNRRLRTIDELACDEIRKGFLKL
RRTVQERMSLKDAADMTPRSLINPKSISAAIEYFFGRGELSQVVDQTNPLSMLTHERRLS
ALGPGGLNRKRAGFEVRDVHISHYGRICPIETPEGTNIGLISSLAMYATVDDYGFLVTPY
RKVKNGKLTEEVVWLRADEESDAYVASADVPVKNNEIQGDTVVARYRSDFMVVPTEKIQY
TDVAPSQMIGVSAGLIPFLEHDDANRALMGSNMQRQAVPLLIAEPPIVGTGLERDVARHS
GMLVRAGHKGTVTYVDADRIEIGPEKHQMRKFVGLNERTCQNQKPLVKIGDKVEKGDVIA
DGAATYRGDLALGRNVLVAFMSYEGYNFEDAIIISEELVHNDTYTSIHIEEFDVEIRETK
LGREEFTRDIPNVSEKALRNLDESGIVRVGTFVWPGDILVGKVSPKSKTELTPEEKLLHA
IFGRAGEDVKNDSLEVPSGVEGIVIDTQKFSRRMSLSEDERKAFERSLKDAEKSGNDKIA
AMFTALATEIEKILGKKLTDEDGNELIHNQEPQFVADQAVNFKVDNLDIRSDDRKKSVQQ
AYKQLWPDVEEAIDERDRTLNSMKRGDELRSGVLQMVKVYVATKRVISVGDKMAGRHGNK
GVISKILPIEDMPFLPDGTPVQIMLNPLGVPSRMNVGQILETHLGWAGAKLGFRAVTPVF
DGATEEEINDCLEKAGLPRHGKARLNDGRTGMPFEQETTVGFIYMLKLHHLVDDKVHARS
TGPYSLITQQPLGGKARFGGQRFGEMEVWALEAYGAAYILQELLTVKSDDVEGRTKIYES
MVKGENTLQAGTPASFDVLTNEIRGLGLNMQLEKRVL
>sp|A5IQ96|RPOB_STAA9 DNA-directed RNA polymerase subunit beta OS=Staphylococcus aureus (strain JH9) OX=359786 GN=rpoB PE=3 SV=1
MAGQVVQYGRHRKRRNYARISEVLELPNLIEIQTKSYEWFLREGLIEMFRDISPIEDFTG
NLSLEFVDYRLGEPKYDLEESKNRDATYAAPLRVKVRLIIKETGEVKEQEVFMGDFPLMT
DTGTFVINGAERVIVSQLVRSPSVYFNEKIDKNGRENYDATIIPNRGAWLEYETDAKDVV
YVRIDRTRKLPLTVLLRALGFSSDQEIVDLLGDNEYLRNTLEKDGTENTEQALLEIYERL
RPGEPPTVENAKSLLYSRFFDPKRYDLASVGRYKTNKKLHLKHRLFNQKLAEPIVNTETG
EIVVEEGTVLDRRKIDEIMDVLESNANSEVFELHGSVIDEPVEIQSIKVYVPNDDEGRTT
TVIGNAFPDSEVKCITPADIIASMSYFFNLLSGIGYTDDIDHLGNRRLRSVGELLQNQFR
IGLSRMERVVRERMSIQDTESITPQQLINIRPVIASIKEFFGSSQLSQFMYQSNPLSDLT
HKRRLSALGPGGLTRERAQMEVRDVHYSHYGRMCPIETPEGPNIGLINSLSSYARVNEFG
FIETPYRKVDLDTHAITDQIDYLTADEEDSYVVAQANSKLDENGRFMDDEVVCRFRGNNT
VMAKEKMDYMDVSPKQVVSAATACIPFLENDDSNRALMGANMQRQAVPLMNPEAPFVGTG
MEHVAARDSGAAITAKHRGRVEHVESNEILVRRLVEENGVEHEGELDRYPLAKFKRSNSG
TCYNQRPIVAVGDVVEYNEILADGPSMELGEMALGRNVVVGFMTWDGYNYEDAVIMSERL
VKDDVYTSIHIEEYESEARDTKLGPEEITRDIPNVSESALKNLDDRGIVYIGAEVKDGDI

LVGKVTPKGVTELTAEERLLHAIFGEKAREVRDTSLRVPHGAGGIVLDVKVFNREEGDDT
LSPGVNQLVRVYIVQKRKIHVGDKMCGRHGNKGVISKIVPEEDMPYLPDGRPIDIMLNPL
GVPSRMNIGQVLELHLGMAAKNLGIHVASPVFDGANDDDVWSTIEEAGMARDGKTVLYDG
RTGEPFDNRISVGVMYMLKLAHMVDDKLHARSTGPYSLVTQQPLGGKAQFGGQRFGEMEV
WALEAYGAAYTLQEILTYKSDDTVGRVKTYEAIVKGENISRPSVPESFRVLMKELQSLGL
DVKVMDEQDNEIEMTDVDDDDVVERKVDLQQNDAPETQKEVTD
>tr|A0A809NEF2|A0A809NEF2_LACRG DNA-directed RNA polymerase subunit beta OS=Lacticaseibacillus rhamnosus (strain ATCC 53103 / LMG 18243 / GG) OX=568703 GN=rpoB PE=3 SV=1
MNYGKHRTRRSYARIKEVLDLPNLIEIQTNSYQWFLDEGLKEMFDDIMPIDDFQGKLSLE
FVGYQLLEPKYTVEEARQHDANYSAPLHVTLRLTNHETGEIKSQDVFFGDFPLMTKQGTF
IINGAERVIVSQLVRSPGVYFHSETDKNSRVTYGTTVIPNRGAWLEYETDAKDIAYVRID
RTRKIPLTELVRALGFGSDQDIINMFGDNDSLMLTLEKDVHKNTDDSRTDEALKDIYERL
RPGEPKTADSSRSLLYARFFDPKRYDLASVGRYKVNKKLSLKTRLLNQVLAETLADPDTG
EVVAQKGTKVDRQVMDKLAPYLDRDDFKTATYQPSDQGVMTDPIELQSIKVYSQVTPDKE
INLIGNGHIGKKVKHILPADVLASMNYFLNLQEGLGTVDDIDHLGNRRIRSVGELLQNQF
RIGLSRMERVVRERMSIQDTATVTPQQLINIRPVVASIKEFFGSSQLSQFMDQTNPLGEL
THKRRLSALGPGGLTRDRAGYEVRDVHYTHYGRMCPIETPEGPNIGLINSLASYAVVNPY
GFIETPYRRVSWDTHKVTDKIDYLTADEEDNYIVAQANSPLNDDGSFVDDTVLARHKDNN
IEISPDKVDYMDVSPKQVVAVATACIPFLENDDSNRALMGANMQRQAVPLINPHAPLVGT
GMEYKAAHDSGTAVLANNAGTVEYVDAKQIRVRREDGALDTYKLMKFKRSNAGKNYNQRP
IVTIGDHVDVDEIIADGPAMQNGELALGQNPIIAFMTWNMYNYEDAIVLSERLVKDDVYT
SIHIEEYESEARDTKLGPEEVTREIPNVGEEALKDLDEFGVVRVGAEVRDGDILVGKVTP
KGVTELSAEERLLHAIFGEKAREVRDTSLRVPHGGGGIIQDVKIFTREAGDELSPGVNMM
VRVYITQKRKIQVGDKMAGRHGNKGTVSVVVPEEDMPYLPDGTPVDICLSPMGVPSRMNI
GQVLELHLGMAARNLGIHVATPVFDGANDKDLWATVKEAGMPSDGKSVLYDGRTGEPFEN
RVSVGVMYYMKLSHMVDDKIHARSIGPYSLVTQQPLGGKAQFGGQRFGEMEVWALEAYGA
AYTLQEILTYKSDDVVGRVKTYEAIVKGDPIPKPGVPESFRVLVKELQALGLDMKVLGAD
KQEIELRDMDDDEDDVVSVDALAKFAAQQEEKKAHEAATQASDGQSADNSTDDKK
>sp|Q8GCR6|RPOB1_ENTFC DNA-directed RNA polymerase subunit beta OS=Enterococcus faecium OX=1352 GN=rpoB PE=3 SV=1
MKSLAGHVVKYGKHRERRSFARISEVLELPNLIEIQTDSYQWFLDEGLREMFEDILPIDD
FNGNLSLEFVDYELKEPKYTVAEARAHDANYSAPLHVTLRLTNRETGEIKAQEVFFGDFP
LMTEQGTFIINGAERVIVSQLVRSPGVYFHGKVDKNGKEGFGSTVIPNRGAWLEMETDAK
DISYVRIDRTRKIPLTVLVRALGFGSDDTIFEIFGDSETLRNTVEKDLHKNASDSRTEEG
LKDVYERLRPGEPKTADSSRNLLNARFFDPKRYDLANVGRYKVNKKLDLKTRLLNLTLAE
TLVDPETGEIIVEKGTVLTHQVMETLAPFIDNGLNSGTYYPSEDGVVTDPMTVQVIKVFS
PRDPEREVNVIGNGYPEAAVKTVRPADIIASMSYFLNLMEGIGNVDDIDHLGNRRIRSVG
ELLQNQFRIGLARMERVVRERMSIQDTETLTPQQLINIRPVVASIKEFFGSSQLSQFMDQ
TNPLGELTHKRRLSALGPGGLTRDRAGYEVRDVHYSHYGRMCPIETPEGPNIGLINSLSS
YAKVNKFGFIETPYRRVDRETGRVTDQIDYLTADIEDHYIVAQANSPLNEDGTFAQDVVM
ARAQSENLEVSIDKVDYMDVSPKQVVAVATACIPFLENDDSNRALMGANMQRQAVPLINP
QAPWVGTGMEYKSAHDSGAALLCKHDGVVEYVDASEIRVRRDNGALDKYDVTKFRRSNSG
TSYNQRPIVHLGEKVEKGVTLADGPSMEQGEMALGQNVLVGFMTWEGYNYEDAIIMSRRL
VKDDVYTSIHIEEYESEARDTKLGPEEITREIPNVGEDALKDLDEMGIIRIGAEVQDGDL
LVGKVTPKGVTELSAEERLLHAIFGEKAREVRDTSLRVPHGGGGIVHDVKIFTREAGDEL
SPGVNMLVRVYIVQKRKIHEGDKMAGRHGNKGVVSRIMPEEDMPFLPDGTPIDIMLNPLG
VPSRMNIGQVLELHLGMAARQLGIHVATPVFDGASDEDVWETVREAGMASDAKTVLYDGR
TGEPFDGRVSVGVMYMIKLAHMVDDKLHARSIGPYSLVTQQPLGGKAQFGGQRFGEMEVW
ALEAYGAAYTLQEILTYKSDDVVGRVKTYEAIVKGEPIPKPGVPESFRVLVKELQSLGLD

MRVLDIEETEIELRDMDDEDDDLITVDALTKFAEQQTAKELEKKAAEQVEDEKDDVIQNF
ETAEDNLD
>tr|A0A9D5IBI1|A0A9D5IBI1_9PLAN DNA-directed RNA polymerase subunit
beta OS=Planctomyces sp OX=37635 GN=rpoB PE=3 SV=1
MADLNVRSYAKRGDVVTIPDLTKIQADGYERFLQTQKGPDERDPQTGLESLMREVYPIES
YDGTMRLEYIRYMLEEPRYTQDECRELRLTYGAPFKVGLRLTRDGQQEVAEEEIYLGEFP
IMLGGGEFIVNGAERVIVSQLHRSPGVDFSIVTDEGDRPLHSARIIPERGSWIELEVTKK
DELVMRIDQSTKIATTTFLRCLDESVSTTEAVLSLFYEISEIKAEQVHADHYAAGAIIDT
ETGEELVPVGRKIGEDAAGKIAGSKLKSLRVIQNPSDPLILNTVAMEKLEQFAENAESEY
ERALLKIYTRLRPGNPPQVEKAKSLFVEKFFDENRYRLGKVGRFRINRKFDLNVPEDMMF
IRAEDFLRVVQYLLDLRSKRLDPKTGFPVAQVDDIDHLGNRRLRTLDELAVEELRKGFLK
LRRTVQERMSVKDPTETIKIADLVNSKSISAAIEFFFGRSELSQVVDQTNPLSSLVHERR
LSALGPGGLNRKRAGFEVRDVHISHYGRICPIETPEGTNIGLIASLGIFSSIDDYGFLRT
PYRVVKDGKVQDKVLPLRADEEMQAILAPTDALEPTGQLRQGLILARVNGELKEVDSSMV
NYVDVGPKQIVGVSAALIPFLEHDDANRALMGSNMQRQAVPLIKVTPSIVATGLEKEIGK
NSGFVVRAKNGGVVTFVDAERIIVDNSDEYVLRKHAGLNERTCQNQRPIVRPGQRVKKGE
VIADGASTRQGELAIGKNILVAFNTYDGYNFEDAIVINERLVKDDVFTSIHIDAFEVEVR
ETKLGREEFTRDIPNVSEKMLRNLDELGIVRLGARVGPGDILVGKVSPKSKTELTPEEKL
LHAIFGRAGEDVKNDSLEVPAGVEGIVIGAKRFSRRMHMNEEQKKALKVQIEEYTREMDD
KAIGIFKQMVAAVNDILGTPMVDPTTRQKVGASDIREVVLEQIESFSDKWLKGSKEAKEK
AEVVYRQFWPRIAAIKAEKKRKTDHMRRGDELPSGVLEMVKVYLATKRHLSVGDKMAGRH
GNKGVIARIVAEEDMPFMDDGSTVDILLNPLGVPSRMNVGQILETHLGWAMNILGMQAVT
PVFDGAFEEEIHSAIDEANRAVEEKAARYEREGRWPQPRELMVRMPRGGKIQLHDGRTGE
PFKQRTTVGVMYMLKLHHLVDDKIHARATGPYSLITQQPLGGKARTGGQRFGEMEVWALE
AYGAAYILQELLTVKSDDIEGRTKIYESMVKGTNTLEAGMPTAFDVLCSELKGLGMNISL
EKKKTQGVGLL
>sp|Q59191|RPOB_BORBU DNA-directed RNA polymerase subunit beta
OS=Borreliella burgdorferi (strain ATCC 35210 / DSM 4680 / CIP
102532 / B31) OX=224326 GN=rpoB PE=3 SV=2
MIKRVHLGQGRADEILDLPNLIEIQLNSYEKFLQLDKLKSKKPLLNEGLESVFRNIFPIK
SGNGDVALEYERYYIENDALNFTEKECKRKGQSYEAVLKVRLNLQFLTTGEIRQKDVYMG
TIPLMTERGTFIINGAERVVVSQIHRSPGVVFYKEKDLYSARIIPYRGSWLEFEIDSKKD
YLYVKIDRKKRILITLFLRALGFDTREKIIETFYNIKKIKVEDGTKRDLPGQYLAKSINI
RENMYYRAGDKITLQDVEDFLQNGVNEIELVDFDGYNDISGKRFVSSNVILNCLEKEDAF
FALKDGSKELPKESVMLAVYGSLFPGEPISIDNAENDLKTIFFSERRYDLGRVGRYKLSK
KFGFDDLTTSVLTMDDIVNTISHLLRIYEGHDILDDIDHLGNRRVRSVGELLTNIYKGAM
SRVEKIAKDRMSNKEVFNLKPQELISVKPIVSAVKEFFATSQLSQFMDQVNPLAELTHKR
RLNALGPGGLSRDRAGFEVRDVHYTHYGRMCPIETPEGPNIGLIVSLATYSRVNDYGFLE
TPYRKVVNGVVTDQLEYLSAIDEEKKCIAQANAAFNSNGKYLEDLVSVRISGDYTTTSPT
NIDYMDVSPRQLISVSSALIPFLEHNDANRALMGSNMQRQAVPLLFPKPPIVGTGMESVV
AKDSGVVVKAKRSGEVILATSSKIVVKPFEAENAKDLDEYHIVKYERTNQDTCFNQSVLV
KEGQKVERGEIIADGPATRYGELALGNNLLLGVIPWNGFNYEDAILISDRIVKEDLYTSI
HIKEFSIEVRETKLGPEKVTGDIPNVSEKILNKLDENGIIRIGTYVKPGDILVGKVTPKS
EGDITPEFRLLTSIFGEKAKDVKNNSLKVPHGTEGTVIDVQRITKEDVGNLSPGVEEILK
VYVAKKRKLKEGDKMAGRHGNKGVVAKILPVEDMPYLADGTPLDICLNPLGVPSRMNIGQ
LMESQLGLAGKYLGESYNVPVFESATNEQIQEKLKTAGFNPTSKEILYDGYTGEPFENEV
MVGVIYMLKLHHLVDDKMHARSTGPYSLVSQQPLGGKAQFGGQRLGEMEVWALEAYGAAH
TLQELLTVKSDDMSGRVKIYENIVKGVPTNVSGIPESFNVLMQELRGLGLDLSIYDDAGN
QVPLTEKEEELINKS
>tr|A0A7M1XI38|A0A7M1XI38_9SPIR DNA-directed RNA polymerase subunit

beta OS=Treponema rectale OX=744512 GN=rpoB PE=3 SV=1
MEELDLNKYERTSTGRIDMSKTFSNVPLPNLCDVQLQSFKWFSETGVDEVFKDIFPIQSN
KDTRGHETDTEQIAELDYVKSEWGNADEKHGYFECKVSALTYSAPLHVTLRLKHPDGTVS
EEKIFMGDFPWITPSGTFIINGSEKCIASQLVRSPGAYVSKEADEVTVKKSDNDAKNEIN
LVYGSDIIPARGIWLEYLTDSRDFVSVRIDKQKKVPALVLLRALGIVSDADSLNDQSLPA
NSDGTASPAVTGVVGLFGENSYLAKALEKNSVNANKGALDRAHEAVGSIFRKLRPGEPYT
AQSAASTLKQRFFENEHYDLGKAGRFKINDKLGVYERLIEQTLAEDLISRDGEIVYEAGH
TLTTEEVIALKRDNFFDNNENHTVILSSNEELDNHNRVNLVKIKNPNDPEKVIHVVGTDL
TVNLTYVTIPDIVASLSYMLNLIDGVGYTDDTDHLGNKRVRCVGELIQDKFRAGLSKMKR
TIHDRMSTSDLASMNISSLINIKALTSAVNQFFNSDSLSQFMDQTNPLAELTNKRRLSAL
GRGGLTRDRASSAVRDVHPTHYGRICPIETPEGQNIGLISNLACYAKVNEYGFLQTPYRP
VHDRIVDNDKDHVLWLTAAEERNHVICQANVVVSKDSNRIEDDTVAARFNGEYITAKAEE
CDLIDASPKQIVSIAAACIPFLENDDGKRALMGSNMQRQALPLLRPEAPYVGTGLEEKIA
HDSGEALLAYKPGYVDYVDSRQIVIIQDDGTKKIYKLRSFVRSNKRTCISQSPKVHVGDH
VEAGDIIANGPSMDKGELALGQNAVVAFTTWHGYNYEDAVVISERCVNQDLFTNLIIEEY
PIERRKTKLGDEEFTRNVPNLGEDKKTYLDEKGIVVPGTEVHEGDILVGKTTPKGEVTET
NNDHLLKSIFSSKSDEDKDTSLRVPHGGEGIVLDVRTFSRDKGDELPPDVLESAKVYVVQ
KRKIQVGDKMSGRHGNKGVISRVLPVEDMPYLPDGTPVDILLSPMGVPSRMNIGQVLEVQ
LGLACKKLGIKVSTPVFDGASNEEIAQFMEKAGIDSDGKTVLYDGQTGERFDSRIAVGVM
YMIKLDHMVEDKIHARAIGPYSLVTQQPLGGKAQNGGQRFGEMEVWALEAYGAAYTLQEM
LTIKSDDMVGRNKTYEAILKGNQVSKPNMPESTRVLIKELQGLGIDVNLLDDKNQPINMD
TISEEAEREGRRTHHDIVNFDTKRDEEDELEDARNAALDEEIDDEFSDNENPETDDFADD
DME
>tr|A0A0E3YV74|A0A0E3YV74_9BACT DNA-directed RNA polymerase subunit
beta OS=Verrucomicrobia bacterium IMCC26134 OX=1637999 GN=rpoB PE=3
SV=1
MADRNQFERLNFGKLREVIQPPNLIEIQITSYLDFLQKGVPDKQRKAQGLEAVFREIFPI
ESYDGRLVLEYVSYTLGDPRNTEIECIKDGITYSVPLHVKLRLREEEFIKDEEIYMGEIP
MVTERGSFIINGAERVVVSQLHRSPGLCFEKDRHTSGKELFAFRIIPDRGTWLEVQFDQN
DLLYVYLDRRRRRRKFLITTLFRSLGYSDNAKILELFYEVKDVKINKLLDLENVSTLVFV
DDVIDAHKGVVLARALEQVTKQTIRTFEKHDITNVRILDTAVDDGAIVRALKKDPTINEE
EALKEIYKRLRPGEPATIANAKALLKRLFFDPKRYDLGAVGRYKINQKLHLETPIEQRIV
TTEDMIASTKYLVRLKRGEGVVDDIDHLGSRRVRTVGELLANECRKGLARTERLVRERMT
MYDQSVDSITPNKLINPKALTTVIRDFFARSQLSQFMDQINPLAEVTHKRRLSALGPGGL
NRDRAGFEVRDVHPSHYGRICPIETPEGPNIGLINSLSTYARVNEYGFIEAPYRLVKDGK
VSDKIEYLTANQEEAKVIAQANAEIDEKGHFVGKVTVRKDGEFLEVSASEVHYMDVSPKQ
VISIAAGMIPFLEHDDANRALMGANMQRQGVPLLQTESPYVGTGIEERVARDSKIVVVAE
ESGYVASVDAKRIVISKDGELPRNFEKNPKSDSKTGVQVYELRKFMRSNAGTNFNQKPIV
KKGQKIKANEIIADGPSTDKGEMALGRNILVAFMPWNGYNFEDAILISEKVLKEDIFSSI
HIQEFEVTARDTKLGPEEITRDIPNVGEEALKNLDHNGVIRIGAEVKPGDILVGKITPKS
ETELAPEEKLLRAIFGEKAADVKDTSLIVPSGVTGIIMDVKVSSRVDFEKEKLSPSDRRR
EIKQIQEEYKTQMDKLRESLTEALSNILLGEKIPLDVTNGATGEIIIPANRKITKTLLRK
LAAVSKHVEIDPSPVRIKIMEIIASFQSRFDELESDRERKVSGIESGDIAGDGSIKQVKV
YIATKQKLEVGDKMAGRHGNKGVVAKIVPEADMPFLPDGTPIQICLNPLGVPSRMNVGQV
LETHLGWACSKLGIKVATPVFDGIPEKKVREYLKEAGLPTSGKSVLFDGRTGDKIDQEVV
VGYIYMMKLNHLVSHKIHARAVGPYSLVTQQPLGGKAQYGGQRFGEMEVWALEAYGAAHT
LQELLTVKSDDVQGRTKIYESLVKGDNTLQAGTPESFNVLIKEIQSLGLDIRLNKRDALG
NLVETRTPSAATIASGNPRATSL
>sp|Q18CF1|RPOB_CLOD6 DNA-directed RNA polymerase subunit beta
OS=Clostridioides difficile (strain 630) OX=272563 GN=rpoB PE=1 SV=1

MPHPVTIGKRTRMSFSKIKEIADVPNLIEIQVDSYEWFLKEGLKEVFDDISPIEDYTGNL
ILEFVDYSLDDKPKYDIEECKERDATYCAPLKVKVRLINKETGEIKEQEVFMGDFPLMTE
RGTFVINGAERVIVSQLVRSPGVYYAEERDKTGKRLISSTVIPNRGAWLEYETDSNDVIS
VRVDRTRKQPVTVLLRALGIGTDAEIIDLLGEDERLSATLEKDNTKTVEEGLVEIYKKLR
PGEPPTVESASSLLNALFFDPKRYDLAKVGRYKFNKKLALCYRIMNKISAEDIINPETGE
VFVKAGEKISYDLAKAIQNAGINVVNLLMDDDKKVRVIGNNFVDIKSHIDFDIDDLNIKE
KVHYPTLKEILDGYSDEEEIKEAIKSRIKELIPKHILLDDIIASISYEFNIFYNIGNIDD
IDHLGNRRIRSVGELLQNQVRIGLSRMERVIKERMTVQDMEAITPQALVNIRPVSAAIKE
FFGSSQLSQFMDQTNPLSELTHKRRLSALGPGGLSRERAGFEVRDVHHSHYGRMCPIETP
EGPNIGLINSLGTYAKINEFGFIESPYRKFDKETSTVTDEIHYLTADEEDLFVRAQANEP
LTEDGKFVNHRVVCRTVNGAVEMVPESRVDYMDISPKQVVSVATAMIPFLENDDANRALM
GANMQRQAVPLVRREAPIIGTGIEYRAAKDSGAVVVARNSGIAERVTADEIIIKREDGNR
DRYNLLKFKRSNSGTCINQTPIINKGDQIIKGDVIADGPATDLGEVALGRNCLIAFMTWE
GYNYEDAILINERLVKEDRLSTIHIEEYECEARDTKLGPEEITRDIPNVGDSAIKNLDDR
GIIRIGAEVDSGDILVGKVTPKGETELTAEERLLRAIFGEKAREVRDTSLKVPHGESGII
VDVKVFTRENGDDLSPGVNELVRCYIAKKRKIKVGDKMAGRHGNKGVISRVLPEEDMPFM
ENGTPLDIILNPQGIPSRMNIGQVLEVHLGLAAKTLGWYVATSVFDGANEYDIMDALEEA
GYPRDGKLTLYDGRTGESFDNRITVGYMYYLKLHHLVDEKLHARSTGPYSLVTQQPLGGK
AQFGGQRFGEMEVWALEAYGAAHILQEILTVKSDDVVGRVRTYEAIVKGENIPEPGIPES
FKVLIKELQSLCLDVKVLTDEDQEIEVRESVDEDDTIGEFELDVVNHMGEVEESNIIEEI
EDDFAENAEDEDIENLEEFTEDDLFEEEIDFDSDDFDM
>sp|P37870|RPOB_BACSU DNA-directed RNA polymerase subunit beta
OS=Bacillus subtilis (strain 168) OX=224308 GN=rpoB PE=1 SV=2
MTGQLVQYGRHRQRRSYARISEVLELPNLIEIQTSSYQWFLDEGLREMFQDISPIEDFTG
NLSLEFIDYSLGEPKYPVEESKERDVTYSAPLRVKVRLINKETGEVKDQDVFMGDFPIMT
DTGTFIINGAERVIVSQLVRSPSVYFSGKVDKNGKKGFTATVIPNRGAWLEYETDAKDVV
YVRIDRTRKLPVTVLLRALGFGSDQEILDLIGENEYLRNTLDKDNTENSDKALLEIYERL
RPGEPPTVENAKSLLDSRFFDPKRYDLANVGRYKINKKLHIKNRLFNQRLAETLVDPETG
EILAEKGQILDRRTLDKVLPYLENGIGFRKLYPNGGVVEDEVTLQSIKIFAPTDQEGEQV
INVIGNAYIEEEIKNITPADIISSISYFFNLLHGVGDTDDIDHLGNRRLRSVGELLQNQF
RIGLSRMERVVRERMSIQDTNTITPQQLINIRPVIASIKEFFGSSQLSQFMDQTNPLAEL
THKRRLSALGPGGLTRERAGMEVRDVHYSHYGRMCPIETPEGPNIGLINSLSSYAKVNRF
GFIETPYRRVDPETGKVTGRIDYLTADEEDNYVVAQANARLDDEGAFIDDSIVARFRGEN
TVVSRNRVDYMDVSPKQVVSAATACIPFLENDDSNRALMGANMQRQAVPLMQPEAPFVGT
GMEYVSGKDSGAAVICKHPGIVERVEAKNVWVRRYEEVDGQKVKGNLDKYSLLKFVRSNQ
GTCYNQRPIVSVGDEVVKGEILADGPSMELGELALGRNVMVGFMTWDGYNYEDAIIMSER
LVKDDVYTSIHIEEYESEARDTKLGPEEITRDIPNVGEDALRNLDDRGIIRIGAEVKDGD
LLVGKVTPKGVTELTAEERLLHAIFGEKAREVRDTSLRVPHGGGGIIHDVKVFNREDGDE
LPPGVNQLVRVYIVQKRKISEGDKMAGRHGNKGVISKILPEEDMPYLPDGTPIDIMLNPL
GVPSRMNIGQVLELHMGMAARYLGIHIASPVFDGAREEDVWETLEEAGMSRDAKTVLYDG
RTGEPFDNRVSVGIMYMIKLAHMVDDKLHARSTGPYSLVTQQPLGGKAQFGGQRFGEMEV
WALEAYGAAYTLQEILTVKSDDVVGRVKTYEAIVKGDNVPEPGVPESFKVLIKELQSLGM
DVKILSGDEEEIEMRDLEDEEDAKQADGLALSGDEEPEETASADVERDVVTKE
>tr|A0A3M1CCR9|A0A3M1CCR9_9BACT DNA-directed RNA polymerase subunit
beta (Fragment) OS=Bdellovibrio sp OX=28201 GN=rpoB PE=3 SV=1
YYHIDHVKVKDGKIYRELDIQKIAGQRALSDIVDPKTGDVLVKAGRRITRAVVNKVKAAN
IKEVEISKEDLAGKVLGRPLIDEETGEIIADVNQELTVDIIDRCLSLGITDFYVIFFDGL
SVGPSLRNTLLIDNVATEEEALIEIYKRLRPGEPPTEEAARNFFHRLFFDPETYDLSEVG
RLKINHRFNISFEECPVDHRTLTKKDILEVVRTLIDLKNGHGEVDDIDHLGNRRVRSVGE
LLENQYRIGLVRMERAIKERMSLQDLETMMPHDLINAKPVNAVVKEFFGSSQLSQFMDQT

NPLSEITHKRRLSALGPGGLTRDRAGFEVRDVHPTHYGRICPIETPEGPNIGLIASLATY
ARINKYGFIETPYREVEHAHVKPDIKYLSALEEAGHYIAQAGKDVKNDEIVADTVTVRVN
GEYEVVEKDKVTLMDVSPSQLVSIAASLIPFLEHDDANRALMGSNMQRQAVPLLRAKAPL
VGTGVEKLVARDSGTSVVCANDGVVEEVDASRIVVRRLAKGGQLGANVDIYNLVKYQRTN
QNTCFNQRPIVKVGDHVKKGDVIADGPSTEYGELALGQNVMVAFTPWMGYNFEDSILISE
RLIRDDVYTSVHVEEFECVARDTKLGKEEITRDIANVGEEALKDLDSSGIIRIGAEVKPG
DILVGKVTPKGETQLSPEEKLLRAIFGEKAGDVKDTSLRVPSGVFGTVIDAQVYSREGAG
RDERLLSIIEGKKKKLEKDMNVERNIIKNNAIDKLKELLVGRKTTGVLLSEDGSEKLLKK
GQEITAEDLETIPFELLAYIPLETDLEYQVSRIVDAARNQLDALKVVFDEKVERLSKGDE
LPPGVIKMVKVYVAIKRKLQVGDKFAGRHGNKGVISRILPIEDMPYLADGTPVDMVLNPL
GVPSRMNIGQILEIHLGWAAHGLGKQLEKYLKHFNAEEARRALKEVYDTEELEKVIDKAD
DDSLRKMIEIVKDGVHLGTPVFDGAKEEDVRKLLRKAQLPETGQTVLFDGRTGEPFQTPV
TVGIMYMLKLHHLVEEKIHARSIGPYSLVSQQPLGGKAQFGGQRLGEMEVWAIEAYGAAY
SLQEFLTVKSDDVVGRTRMYESIVKGENILEPGLPESFNVLVKELKSLALNVELIESDVL
TEDPEDISGAVEDIEQ
>tr|A0A7W8FG20|A0A7W8FG20_9BACT DNA-directed RNA polymerase subunit beta OS=Desulfovibrio intestinalis OX=58621 GN=rpoB PE=3 SV=1
MGQLTKQFGKIKISLPIPHLLNLQIDSYEKFLQEGVPEADRRPDEGLEGVFHTVFPIEDF
NKTASLEFVSYEVGEPKYDQAECISKGLTYEAPMRIKVRLVVYDADEASGNRTIRDIKEQ
DIYFGTLPLMTEKGTFIINGTERVIVNQLQRSPGIIFEHDGGKTHTSRKVLYSCRVIPMR
GSWLDFDFDHKDILYVRIDRRRKMPATILFKAMGMSKEQILDYFYSRETYRLEDRNAIYW
EVRKELYRKDNAYADIVDGEGNVIVKAGKPITKRSWRLICDAGIEAIEMRPDTLDSMFLA
VDVADPKTGELLAEAADEITPGLLDRIREAGIDRVSVLHTKGNDTSSSIRDTLMLDRIPD
QQKAQEEIYRRLRPSSPPTAEIAASFFDNLFRNGDYYDLSPVGRYKLNQRLALDESSDLR
TLTDNDILTAIKVLVNLKDSHGPADDIDHLGNRRVRLVGELVENQYRIGLVRMERAIKER
MSLQEISTLMPHDLINPKPVAAVLKEFFGTSQLSQFMDQTNSLSEVTHKRRLSALGPGGL
TRERAGFEVRDVHTSHYGRICPIETPEGPNIGLIVSLTTFAKVNDFGFIETPYRVVREAR
VTEDVVHLDASREGDQVIAQANALVDEDGNLLDEFVTVRVKGEVEMRHRDEVNLMDISPS
QMVSISAALIPFLEHDDANRALMGSNMQRQAVPLLRSEKPLVGTGMEVDVARDSGACIVA
PADGKVEYVDADRIVVAYEGEVYKKQGGVRAYDLLKYHKSNQNSCFGQKPTCRPGQLVKK
GQILADGPGIDDGELALGKNLVVAFMPWCGYNYEDSILISERTVKEDVFTSIHIEEFEVV
ARDTKLGPEEITRDIPNVSEDMLRNLDESGIIRIGAAVKPDDILVGKITPKGETQLTPEE
KLLRAIFGEKARDVKNTSLKVPPGVEGTIIDVKVFNRRSGEKDDRTLAIEVHDTSVLDQK
EADHLRALTDRTRVLLTPHVNGKQVAASVPGKKKGEVLVEAGAALTEDMLADLPVKKLAG
LFKSKEVNDAVADILKSYDQQVDYLRAIYDSKREKVTEGDDLPPGVIKMVKVHIAIKRKL
SVGDKMAGRHGNKGVVSCILPEEDMPFFADGRPVDIVLNPLGVPSRMNIGQIMETHLGWG
AKELGRQLAELLDSGAAMQVLRDEVKNVYSSNEISTLVDAMDDEEFKASVLKLRNGIVTK
TPVFDGATEEEIWGWMEKAGIANDGKTTLYDGRTGEAFKNRVTTGVMYMLKLHHLVDEKI
HARSTGPYSLVTQQPLGGKAQFGGQRLGEMEVWALEAYGAAYLLQEFLTVKSDDVTGRVK
MYEKIVKGDNFLEAGLPESFNVLVKELMSLGLNVTLHQEEGKKKPKRVGYMREREDEA
>sp|Q3SLQ6|RPOB_THIDA DNA-directed RNA polymerase subunit beta OS=Thiobacillus denitrificans (strain ATCC 25259) OX=292415 GN=rpoB PE=3 SV=1
MAYTFTEKKRLRKSFASRTNTLPVPFLLATQLESYRAFLQEGRSRDERLNEGLQAAFTSI
FPIESHSKNARLEYVSYQLGEPVFDIKECQQRGLTYCAPLRAKVRLVIMDKEASKPTIKE
VKEQEVYMGEIPLMTPTGSFVINGTERVIVSQLHRSPGVFFEHDRGKTHSSGKLLFSARV
IPYRGSWLDFEFDAKDILFFRVDRRRKMPVTILLKALGYTPESILDAFFNKDTFYINTLG
IQFELVPERLRGEIARFDICGKDDKVIVAKDKRITAKHIRELDAAGVKKWAVPGEFVVGR
VLAHDVVDKDTGELVARANDEITEELLKKLAAAGIDKFQTLYTNDLDHGAFISQTLRTDD
TTDEYAAKVAIYRMMRPGEPPTEDAVNALFVNLFFSDERYDLSAVGRMKFNRRIGRDELT

GTGTLSTEDIVAVIKILVELRNGRGEIDDIDHLGNRRVRSVGELAENQFRAGLVRVERAV
RERLSQAESDNLMPHDLINAKPVSAAIKEFFGSSQLSQFMDQTNPLSEITHKRRVSALGP
GGLTRERAGFEVRDVHPTHYGRVCPIETPEGPNIGLINSLALFARTNWYGFIETPYRKVV
DNKVTDEIEYLSAIEESKYVIAQANAELDAKGKFKDDLVSCRFKNEFTLSAPDRIEYMDV
APAQIVSVAASLIPFLEHDDANRALMGSNMQRQAVPCLRPEKPFVGTGIERTAAVDSGTV
VIAHRGGRVDYVDAGRIVIRVHDEEARAGEVGVDIYSLIKYTRSNQNTNINQRPLVKVGD
VIARGDVIADGASTDMGELALGQNMLVAFMPWNGYNFEDSILINEKVVAEDRYTSIHIEE
LSVVARDTKLGPEEITRDISNLAERQLARLDESGIIAIGAEVEAGDVLVGKVTPKGETQL
TPEEKLLRAIFGEKASDVKDTSLKVPSGMSGVVIDVQVFTREGIERDKRAQSIIDTQLAE
YKKDLTDRMRIVEDDTFARLEKLLHLKVVNGGPKKLAKGSKISKEYLDSLNRHDWFDIRL
ADDEASRQVESLKDSLTQKRVEFDAMFEEKKQKLTAGDELPPGVQKMVKVYLAVKRRLQP
GDKMAGRHGNKGVVSKIVPVEDMPFMADGRPVDIVLNPLGVPSRMNIGQILETHLGWASK
GLGEKIGQMMAAQTKSVVKELRHFFKEVYNQSGKAEDIDNFTDDEILELGQNLQKGVPFA
SPVFDGATEEEIRRMLALAGLPEGGQVTLYDGRTGEAFDRQVTVGYKHVLKLHHLVDDKM
HARSTGPYSLVTQQPLGGKAQFGGQRFGEMEVWALEAYGASYVLQEMLTVKSDDVNGRTK
VYENIVKGDHKIEAGMPESFNVLVKEIRSLGIDIDLERY
>sp|A0K3L7|RPOB_BURCH DNA-directed RNA polymerase subunit beta
OS=Burkholderia cenocepacia (strain HI2424) OX=331272 GN=rpoB PE=3
SV=1
MQYSFTEKKRIRKSFAKRPIVHQVPFLLATQLESFSTFLQADVPATQRKPEGLQAAFTSV
FPIVSHNGFARLEFVSYALSSPAFNIKECQQRGLTYCSALRAKVRLVILDKESPNKPVVK
EVKEQEVYMGEIPLMTPTGSFVINGTERVIVSQLHRSPGVFFEHDKGKTHSSGKLLFSAR
IIPYRGSWLDFEFDPKDILYFRVDRRRKMPVTILLKAIGLTPEQILANFFVFDNFTLMDE
GAQLEFVPERLRGEVARFDITDRDGKVIVQKDKRINAKHIRDLEAAKTKFISVPEDYLLG
RVLAKNVVDGDTGEVIASANDEVTESVLEKLREAGIKDIQTLYTNDLDQGPYISSTLRVD
ETTDKTAARIAIYRMMRPGEPPTEEAVEALFNRLFYSEEAYDLSKVGRMKFNRRVGRDEI
VGPMTLQDDDILATIKILVELRNGKGEVDDIDHLGNRRVRCVGELAENQFRAGLVRVERA
VKERLGQAESENLMPHDLINSKPISSAIREFFGSSQLSQFMDQTNPLSEITHKRRVSALG
PGGLTRERAGFEVRDVHPTHYGRVCPIETPEGPNIGLINSLALYAHLNEYGFLETPYRKV
VDSKVTDQIDYLSAIEEGRYMIAQANAAIDENGQLIDELVSSREAGETMMVTPDRIQYMD
VAPSQIVSVAASLIPFLEHDDANRALMGSNMQRQAVPCLRPEKPVVGTGIERTCAVDSGT
TVQAFRGGVVDYVDAGRIVIRVNDDEAVAGEVGVDIYNLIKYTRSNQNTNINQRPIVKMG
DKVSRGDVLADGASTDLGELALGQNMLIAFMPWNGYNFEDSILISEKVVADDRYTSIHIE
ELNVVARDTKLGPEEITRDISNLAEVQLGRLDESGIVYIGAEVEAGDVLVGKVTPKGETQ
LTPEEKLLRAIFGEKASDVKDTSLRVPSGMSGTVIDVQVFTREGIQRDKRAQQIIDDELK
RYRLDLNDQLRIVEGDAFQRLARMLVGKVANGGPKKLAKGTKIDQAYLEDLDHYHWFDIR
LADDEAAAQLEAIKNSIEEKRHQFDLAFEEKRKKLTQGDELPPGVLKMVKVYLAVKRRLQ
PGDKMAGRHGNKGVVSKIVPIEDMPYMADGRPADVVLNPLGVPSRMNVGQVLEVHLGWAA
KGLGWRIGEMLQRQAKIEELRTFLTKIYNESGRQEDLESFTDDEILELAKNLREGVPFAT
PVFDGATEEEMGKMLDLAFPDDIAEQLGMNPSKNQVRLYDGRTGEMFERRVTLGYMHYLK
LHHLVDDKMHARSTGPYSLVTQQPLGGKAQFGGQRFGEMEVWALEAYGASYVLQEMLTVK
SDDVTGRTKVYENLVKGDHVIDAGMPESFNVLVKEIRSLGIDIDLDRN
>sp|O52271|RPOB_RICPR DNA-directed RNA polymerase subunit beta
OS=Rickettsia prowazekii (strain Madrid E) OX=272947 GN=rpoB PE=3 SV=1
MVSLRDNIESQPLSHNRRIRKNFGHINLVADIPNLIEIQKNSYEKNFLQLNIKDSERKNK
GLQSILNSIFPISDSSNIANLEFVKYEFDTPKYDVDECSQRSLSYAAPLKVTLRLSIWDI
DEDTGTREIKGIKEQEVYMGDIPLMTKNGTFIINGTERVVVSQMHRSPGVFFYHDEGKVH
SSGKLLYSARVIPYRGSWLDFEFDAKDIIYFRIDRKRKLYATTLLRAIGMSTEEIIKFYY
NSVTYKFVKNKGWSVKFIPQHITAHRLTSDLVDADTGNVLLKAGQKITPRLAQKYFGVGL
NNILVTHETLIGKYLSEDLRDPASDEVLAKIGEMITSDMLKVINDLKIKNVNVLVINPQS

GSYIRNTLFADKNQDREAALCDIFRVLRPGEPANIEAAESLFYNLFFDAERYDLSEVGRI
KMNSRLELNISEEVTVLTIDDIKNIVRILVELKDGKGIIDDIDHLGNRRVRSVGELIENQ
FRIGLVRMEKSVIERMSAGDVDTVMPHDLVNSKILVSVVKEFFSTSQLSQFMDQTNPLSE
ITHKRRLSALGPGGLSRDRAGFEVRDVHPTHYGRICPIETPEGQNIGLINSMATYARINK
HGFIESPYRRVKNGYVTDEVVYLSAIEEGKYKIGQANSKVDQDGKLQGEFINCRVEGGNF
VMVEPDEVDFIDVTPMQVVSVAASLIPFLENDDANRALMGSNMQRQAVPLIKTEAPFVGT
GVEGVVAKDSGASVLALHDGIVERVDSNRIVIRTLEQKVDGSPSVDIYNLLKFQKSNHNT
CINQKPLVKVGHYVKKNDIIADGPSTDNGEIALGRNVLVAFLPWNGYNFEDSILISERIV
KEDVFTSIHIEEFEVIARDTRLGPEEITRDIPNVSEEALRHLDEVGIIYVGAEVKAGDIL
VGKVTPKSESPITPEEKLLRAIFGEKAFDVKDSSLHVPSGVSGTVVEVRIFSRRGVEKDQ
RAIAIEKQQIEKLAKDRDDELEIIEHFVFSWLEKLLVGHVIINGPKQITAGQTITTEMLK
GLSKGQLWQITVEDANVMNEIEQIKIHYDEKKYALDKRFTTKVEKLQSGDDLPQGALKVV
KVFIATKHKLQPGDKMAGRHGNKGVISRIVPEEDMPFLEDGTVVDIVLNPLGLPSRMNIG
QILETHLGWASINLAKKISTLVKEYKDNHIDIEEIKKFLLELYGKDINYILEGSEEEIIS
FCNKVSKGVYFATPVFDGAKVQDVKDMLELAGQDLSGQVKLIDGRTGEYFDRLVTVGHKY
LLKLHHLVDNKIHSRSIGPYSLVTQQPLGGKSHFGGQRFGEMECWALQAYGAAYTLQEML
TVKSDDVNGRIKTYDSIVRGENNFESGIPESFNVMIKEFRSLCLNVKLEVTPSK
>tr|A0A546XZ13|A0A546XZ13_RHIRD DNA-directed RNA polymerase subunit
beta OS=Rhizobium radiobacter OX=358 GN=rpoB PE=3 SV=1
MAQTLSFNGRRRVRKFFGKIPEVAEMPNLIEVQKASYDQFLMVDEPKGGRPDEGLNAVFK
SVFPITDFSGASMLEFVSYEFEAPKFDVEECRQRDLTYAAPLKVTLRLIVFDIDEDTGAK
SIKDIKEQSVYMGDMPLMTNNGTFIVNGTERVIVSQMHRSPGVFFDHDKGKSHSSGKLLF
AARVIPYRGSWLDIEFDAKDIVYARIDRRRKLPVTSLLMALGMDGEDILSTFYTKASYER
DGDGWRIPFQPETLKNAKVVTDMIDADTGEVVVEAGKKLTPRLIRQLSEKGLKALKATDE
DLYGNYLAEDIVNYETGEIYLEAGDEIDEKTLGLILQSGFDEIPVLNIDHVNVGAYIRNT
LSADKNENRQEALFDIYRVMRPGEPPTMESAEAMFNSLFFDAERYDLSAVGRVKMNMRLD
LDAEDTVRTLRKEDILAVVKMLVELRDGKGEIDDIDNLGNRRVRSVGELMENQYRLGLLR
MERAIKERMSSIEIDTVMPQDLINAKPAAAAVREFFGSSQLSQFMDQVNPLSEITHKRRL
SALGPGGLTRERAGFEVRDVHPTHYGRICPIETPEGPNIGLINSLATFARVNKYGFIESP
YRKIVDGKVTNDVIYLSAMEEAKYYVAQANAPLNDDGSFSEEFVVSRHSGEVMLAPRDNI
NLMDVSPKQLVSVAAALIPFLENDDANRALMGSNMQRQAVPLLRAEAPFVGTGMEPIVAR
DSGAAIAARRGGVVDQVDATRIVIRATEDLDAGKSGVDIYRLQKFQRSNQNTCVNQRPLV
SVGDAISKGDIIADGPSTDLGDLALGRNALVAFMPWNGYNYEDSILMSERIVSDDVFTSI
HIEEFEVMARDTKLGPEEITRDIPNVSEEALKNLDEAGIVYIGAEVQPGDILVGKITPKG
ESPMTPEEKLLRAIFGEKASDVRDTSMRMPPGTFGTVVEVRVFNRHGVEKDERAMAIERE
EIERLAKDRDDEQAILDRNVYGRLIDMLRGHVSIAGPKGFKKGVELSNAVVSEYPRSQWW
MFAVEDEKAQSELEALRGQYDESKSRLEQRFMDKVEKVQRGDEMPPGVMKMVKVFVAVKR
KIQPGDKMAGRHGNKGVVSRIVPVEDMPFLEDGTHVDICLNPLGVPSRMNVGQILETHLA
WACAGMGKKIGEMLEEYRKTMDISELRSELTEIYASEANDEVQRFDDDSLVKLAEEAKRG
VSIATPVFDGAHEPDVAAMLKKAGLHESGQSVLYDGRTGEPFDRKVTVGYMYMIKLNHLV
DDKIHARSIGPYSLVTQQPLGGKAQFGGQRFGEMEVWALEAYGAAYTLQEMLTVKSDDVA
GRTKVYEAIVRGDDTFEAGIPESFNVLVKEMRSLGLSVELENSKIENQPEDQLPDAAE
>sp|Q92QH7|RPOB_RHIME DNA-directed RNA polymerase subunit beta
OS=Rhizobium meliloti (strain 1021) OX=266834 GN=rpoB PE=3 SV=1
MAQTLSFNGRRRVRKFFGKIPEVAEMPNLIEVQKASYDQFLMVDEPQGGRPDEGLQAVFK
SVFPIKDFSGASMLEFVSYEFEAPKFDVEECRQRDLTYAAPLKVTLRLIVFDIDEDTGAK
SIKDIKEQNVYMGDMPLMTDNGTFIVNGTERVIVSQMHRSPGVFFDHDKGKSHSSGKLLF
AARVIPYRGSWLDIEFDAKDIVHARIDRRRKIPVTSLLMALGMDGEEILDTFYTKSLYQR
DGEGWRVPFQPDALKGQKTLADMIDADTGEVVVESGKKLTPRLLRQLQEKGLKALKATDD
DLYGNYLAEDVVNFETGEIYLEAGDEIDEKTLPVILSAGFDEIPVLDIDHINIGAYIRNT

LSADKNENRQDALFDIYRVMRPGEPPTMDSAEAMFNALFFDAERYDLSAVGRVKMNMRLD
LDVPDTVRTLRKEDILAVVKMLVELRDGKGEIDDIDNLGNRRVRSVGELMENQYRLGLLR
MERAIKERMSSIEIDTVMPQDLINAKPAAAAVREFFGSSQLSQFMDQVNPLSEITHKRRL
SALGPGGLTRERAGFEVRDVHPTHYGRICPIETPEGPNIGLINSLATFARVNKYGFIESP
YRKIVDGKVTNDVVYLSAMEEAKYHVAQANSVLDDDGSFSEEFVVCRHAGEVMLAPRDNI
NLMDVSPKQLVSVAAALIPFLENDDANRALMGSNMQRQAVPLLRAEAPFVGTGMEPVVAR
DSGAAIAARRGGIVDQVDATRIVIRATEDLDPSKSGVDIYRLQKFQRSNQNTCVNQRPLV
TVGDVINKGDIIADGPSTDLGDLALGRNALVAFMPWNGYNYEDSILLSERIVRDDVFTSI
HIEEFEVMARDTKLGPEEITRDIPNVSEEALKNLDEAGIVYIGAEVQPGDILVGKITPKG
ESPMTPEEKLLRAIFGEKASDVRDTSMRMPPGTFGTVVEVRVFNRHGVEKDERAMAIERE
EIERLAKDRDDEQAILDRNVYARLVDMLRGHVAVAGPKGFKKGTELSNVVISEYPRSQWW
MFAIEDEKAQGEIEALRAQYDESKSRLEQRFMDKVEKVQRGDEMPPGVMKMVKVFVAVKR
KIQPGDKMAGRHGNKGVVSRIVPIEDMPFLEDGTHVDVVLNPLGVPSRMNVGQILETHLG
WACAGMGKKIGAMLDAYKAGADIQPLRDTIDSVIGSGPKGEPIKQYDDESIVRLAEQTRR
GVSIATPVFDGAVEADVNEMLEQAGLKVTGQSTLYDGRTGETFDRQVTVGYIYMLKLNHL
VDDKIHARSIGPYSLVTQQPLGGKAQFGGQRFGEMEVWALEAYGAAYTLQEMLTVKSDDV
AGRTKVYEAIVRGDDTFEAGIPESFNVLVKEMRSLGLSVELENSKVDDVGSTAQLPDAAE
>sp|Q9KV30|RPOB_VIBCH DNA-directed RNA polymerase subunit beta
OS=Vibrio cholerae serotype O1 (strain ATCC 39315 / El Tor Inaba
N16961) OX=243277 GN=rpoB PE=1 SV=2
MVYSYTEKKRIRKDFGTRPQVLDIPYLLSIQLDSFEKFIEQDPEGQYGLEAAFRSVFPIQ
SYNGNSELQYVSYRLGEPVFDVKECQIRGVTYSKPLRVKLRLVIFDKDAPAGTVKDIKEQ
EVYMGEIPLMTENGTFVINGTERVIVSQLHRSPGVFFDSDKGKTHSSGKVLYNARIIPYR
GSWLDFEFDPKDNLYVRIDRRRKLPASIILRALGKTSAEILDIFFEKVNFEVKDQTLMME
LVPERLRGETATFDIEADGKVYVEKGRRVTARHIRQLEKDGVNFIEVPVEYIVGKVSAKD
YVNEATGELIITANQEISLEALANLSQAGYKKLEVLFTNDLDHGPFMSETLRVDSTTDRI
SALVEIYRMMRPGEPPTKEAAESLFESLFFSAERYDLSTVGRMKFNSSIGREDAEEQGTL
DEVDIIEVMKKLISIRNGKGEVDDIDHLGNRRIRSVGEMAENQFRVGLVRVERAVKERLS
LGDLDNVMPQDLINAKPISAAVKEFFGSSQLSQFMDQNNPLSEVTHKRRISALGPGGLTR
ERAGFEVRDVHVTHYGRLCPIETPEGPNIGLINSLSAFARCNEYGFLETPYRRVVNGVVT
DEVDYLSAIEEGQFVIAQANAKLTEEGGFADELVTARQKGESGLHPREHVDYMDVATNQV
VSIAASLIPFLEHDDANRALMGANMQRQAVPTLRSEKPLVGTGIERNVAVDSGVTAVAKR
GGVIQSVDASRIVVKVNEEELIPGEAGIDIYNLTKYTRSNQNTCINQRPCVMPGEPVARG
DVLADGPSTDLGELALGQNMRIAFMPWNGYNFEDSILVSERVVQDDRFTTIHIQELSCVA
RDTKLGAEEITADIPNVGEAALSKLDESGIVYIGAEVKGGDILVGKVTPKGETQLTPEEK
LLRAIFGEKASDVKDTSLRVPNSVAGTVIDVQVFTRDGVEKDKRALEIEQMQLKEAKKDL
TEEFQILEGGLLARVRSVLLAGGYTEAKLGSIERKKWLEQTLENEELQNQLEQLAEQYDE
LKADFDKKFEAKRRKITQGDDLAPGVLKIVKVYLAVKRRIQPGDKMAGRHGNKGVISKIN
PVEDMPYDENGQPVDIVLNPLGVPSRMNIGQILEVHLGLAAKGIGDKINQMIKEQQELAK
LREFLQKVYDLGDTRQRVDISELSDEDVRTLAHNLRAGLPVATPVFDGAPESSIKAMLEL
ADLPASGQLTLFDGRTGDAFERPVTVGYMYMLKLNHLVDDKMHARSTGSYSLVTQQPLGG
KAQFGGQRFGEMEVWALEAYGAAYTLQEMLTVKSDDVNGRTKMYKNIVDGNHAMEPGMPE
SFNVLLKEIRSLGINIELEDE
>tr|A0A7D5W6K4|A0A7D5W6K4_PROMI DNA-directed RNA polymerase subunit
beta OS=Proteus mirabilis OX=584 GN=rpoB PE=3 SV=1
MVYSYTEKKRIRKDFGKRPQVLDVPYLLSIQLDSFQKFIEQDPDGQNGLEAAFRSVFPIQ
SYSGNAELQYVSYRLGEPVFDVKECQIRGVTYSAPLRVKLRLVIYEREAPEGTVKDIKEQ
EVYMGEIPLMTDNGTFVINGTERVIVSQLHRSPGVFFDSDKGKTHSSGKVLYNARIIPYR
GSWLDFEFDPKDNLFVRIDRRRKLPATIILRAMNYSTEDILNLFFEKTTFEISNNKLMMT
LVPERLRGETASFDIEANGKVYVEKGRRITARHIRQLEKEQIERIEVPVEYIAGKVVARD

YIDEATGELICAANMEISLDVLARLSQAGHKTIETLFTNDLDHGAYISETIRVDPTNDRL
SALVEIYRMMRPGEPPTREAAENLFENLFFSEDRYDLSAVGRMKFNRSLGREEVEGSGIL
SQEDIIEVMKKLIDIRNGKGEVDDIDHLGNRRIRSVGEMAENQFRVGLVRVERAVKERLS
LGDLDALMPQDMINAKPISAAVKEFFGSSQLSQFMVQNNPLSEITHKRRISALGPGGLTR
ERAGFEVRDVHPTHYGRVCPIETPEGPNIGLINSLSVYAQTNEYGFLETPYRVVENNAVT
DEIHYLSAIEEGNFIIAQANSVLDDDGHFVEELVTCRHKGESSLFSRDQVQYMDVSTQQV
VSVGASLIPFLEHDDANRALMGANMQRQAVPTLRGDKPLVGTGMERAVAVDSGVTAVAKR
GGTVQYVDASRIVIKVNEDETYAGEAGIDIYSLTKYTRSNQNTCINQTPCVSLGEPVERG
DVLADGPSTDLGELALGQNMRVAFMPWNGYNYEDSILVSERVVQEDRFTTIHIQELACVS
RDTKLGPEEITADIPNVGEAALSKLDESGIVYIGAEVKGGDILVGKVTPKGETQLTPEEK
LLRAIFGEKASDVKDSSLRVPNGVSGTVIDVQVFTRDGVEKDKRALEIEESQLREVKKDL
TEELRIFEAGLFARIRGVLIAGGIEADKLDKLPRERWLELGLADEEKQNQLEQLAEQYDE
LKAEFAKKLEAKRRKITQGDDLAPGVLKIVKVYLAVKRQIQPGDKMAGRHGNKGVISKIN
PIEDMPYDENGNPVDLVLNPLGVPSRMNIGQILETHLGMAAKGIGDKINAMLKQQQEVAK
LREFIQKAYDLGMAPRQKVDLDTFSDEEVLRLAENLKKGMPTATPVFDGAEEMEIKEMLK
LADLPTSGQITLFDGRTGEQFERPVTVGYMYMLKLNHLVDDKMHARSTGSYSLVTQQPLG
GKAQFGGQRFGEMEVWALEAYGAAYTLQEMLTVKSDDVNGRTKMYKNIVDGNHQMEPGMP
ESFNVLLKEIRSLGINIELEDE
>sp|Q8Z320|RPOB_SALTI DNA-directed RNA polymerase subunit beta
OS=Salmonella typhi OX=90370 GN=rpoB PE=3 SV=1
MVYSYTEKKRIRKDFGKRPQVLDVPYLLSIQLDSFQKFIEQDPEGQYGLEAAFRSVFPIQ
SYSGNSELQYVSYRLGEPVFDVQECQIRGVTYSAPLRVKLRLVIYEREAPEGTVKDIKEQ
EVYMGEIPLMTDNGTFVINGTERVIVSQLHRSPGVFFDSDKGKTHSSGKVLYNARIIPYR
GSWLDFEFDPKDNLFVRIDRRRKLPATIILRALNYTTEQILDLFFEKVVFEIRDNKLQME
LIPERLRGETASFDIEANGKVYVEKGRRITARHIRQLEKDDIKHIEVPVEYIAGKVVSKD
YVDESTGELICAANMELSLDLLAKLSQSGHKRIETLFTNDLDHGPYISETVRVDPTNDRL
SALVEIYRMMRPGEPPTREAAESLFENLFFSEDRYDLSAVGRMKFNRSLLRDEIEGSGIL
SKDDIIDVMKKLIDIRNGKGEVDDIDHLGNRRIRSVGEMAENQFRVGLVRVERAVKERLS
LGDLDTLMPQDMINAKPISAAVKEFFGSSQLSQFMDQNNPLSEITHKRRISALGPGGLTR
ERAGFEVRDVHPTHYGRVCPIETPEGPNIGLINSLSVYAQTNEYGFLETPYRRVVDGVVT
DEIHYLSAIEEGNYVIAQANSNLDDEGHFVEDLVTCRSKGESSLFSRDQVDYMDVSTQQV
VSVGASLIPFLEHDDANRALMGANMQRQAVPTLRADKPLVGTGMERAVAVDSGVTAVAKR
GGTVQYVDASRIVIKVNEDEMYPGEAGIDIYNLTKYTRSNQNTCINQMPCVSLGEPVERG
DVLADGPSTDLGELALGQNMRVAFMPWNGYNFEDSILVSERVVQEDRFTTIHIQELACVS
RDTKLGPEEITADIPNVGEAALSKLDESGIVYIGAEVTGGDILVGKVTPKGETQLTPEEK
LLRAIFGEKASDVKDSFLRVPNGVSGTVIDVQVFTRDGVEKDKRALEIEEMQLKQAKKDL
SEELQILEAGLFSRIRAVLVSGGVEAEKLDKLPRDRWLELGLTDEEKQNQLEQLAEQYDE
LKHEFEKKLEAKRRKITQGDDLAPGVLKIVKVYLAVKRRIQPGDKMAGRHGNKGVISKIN
PIEDMPYDENGTPVDIVLNPLGVPSRMNIGQILETHLGMAAKGIGDKINAMLKQQQEVAK
LREFIQRAYDLGADVRQKVDLSTFSDDEVLRLAENLRKGMPIATPVFDGAKEAEIKELLK
LGDLPTSGQITLFDGRTGEQFERPVTVGYMYMLKLNHLVDDKMHARSTGSYSLVTQQPLG
GKAQFGGQRFGEMEVWALEAYGAAYTLQEMLTVKSDDVNGRTKMYKNIVDGNHQMEPGMP
ESFNVLLKEIRSLGINIELEDE
>sp|A6UZI1|RPOB_PSEA7 DNA-directed RNA polymerase subunit beta
OS=Pseudomonas aeruginosa (strain PA7) OX=381754 GN=rpoB PE=3 SV=1
MAYSYTEKKRIRKDFSKLPDVMDVPYLLAIQLDSYREFLQAGATKEQFRDIGLHAAFKSV
FPIISYSGNAALEYVGYRLGEPAFDVKECVLRGVTFAVPLRVKVRLIIFDRESSNKAIKD
IKEQEVYMGEIPLMTENGTFIINGTERVIVSQLHRSPGVFFDHDRGKTHSSGKLLYSARI
IPYRGSWLDFEFDPKDCVFVRIDRRRKLPASVLLRALGYSTEEILNAFYATNVFHIKGET
LNLELVPQRLRGEVASIDIKDGSGKVIVEQGRRITARHINQLEKAGVTQLEVPFDYLIGR

TIAKAIVHPATGEIIAECNTELTLDLLAKVAKAQVVRIETLYTNDIDCGPFISDTLKIDN
TSNQLEALVEIYRMMRPGEPPTKEAAETLFGNLFFSAERYDLSAVGRMKFNRRIGRTEIE
GPGVLSKEDIIDVLKTLVDIRNGKGIVDDIDHLGNRRVRCVGEMAENQFRVGLVRVERAV
KERLSMAESEGLMPQDLINAKPVAAAIKEFFGSSQLSQFMDQNNPLSEITHKRRVSALGP
GGLTRERAGFEVRDVHPTHYGRVCPIETPEGPNIGLINSLATYARTNKYGFLESPYRVVK
DSLVTDEIVFLSAIEEADHVIAQASATLNEKGQLVDELVAVRHLNEFTVKAPEDVTLMDV
SPKQVVSVAASLIPFLEHDDANRALMGSNMQRQAVPTLRADKPLVGTGMERNVARDSGVC
VVARRGGVIDSVDASRVVVRVADDEVETGEAGVDIYNLTKYTRSNQNTCINQRPLVSKGD
VVARGDILADGPSTDMGELALGQNMRVAFMPWNGFNFEDSICLSERVVQEDRFTTIHIQE
LTCVARDTKLGPEEITADIPNVGEAALNKLDEAGIVYVGAEVQAGDILVGKVTPKGETQL
TPEEKLLRAIFGEKASDVKDTSLRVPTGTKGTVIDVQVFTRDGVERDSRALSIEKMQLDQ
IRKDLNEEFRIVEGATFERLRAALVGAKAEGGPALKKGTEITDDYLDGLERGQWFKLRMA
DDALNEQLEKAQAYISDRRQLLDDKFEDKKRKLQQGDDLAPGVLKIVKVYLAIKRRIQPG
DKMAGRHGNKGVVSVIMPVEDMPHDANGTPVDIVLNPLGVPSRMNVGQILETHLGLAAKG
LGEKINRMLEEQRKVAELRKFLHEIYNEIGGREENLDELGDNEILALAKNLRGGVPMATP
VFDGAKEREIKAMLKLADLPESGQMRLFDGRTGNQFERPTTVGYMYMLKLNHLVDDKMHA
RSTGSYSLVTQQPLGGKAQFGGQRFGEMEVWALEAYGAAYTLQEMLTVKSDDVNGRTKMY
KNIVDGDHRMEAGMPESFNVLIKEIRSLGIDIELETE
>sp|P0A8V2|RPOB_ECOLI DNA-directed RNA polymerase subunit beta
OS=Escherichia coli (strain K12) OX=83333 GN=rpoB PE=1 SV=1
MVYSYTEKKRIRKDFGKRPQVLDVPYLLSIQLDSFQKFIEQDPEGQYGLEAAFRSVFPIQ
SYSGNSELQYVSYRLGEPVFDVQECQIRGVTYSAPLRVKLRLVIYEREAPEGTVKDIKEQ
EVYMGEIPLMTDNGTFVINGTERVIVSQLHRSPGVFFDSDKGKTHSSGKVLYNARIIPYR
GSWLDFEFDPKDNLFVRIDRRRKLPATIILRALNYTTEQILDLFFEKVIFEIRDNKLQME
LVPERLRGETASFDIEANGKVYVEKGRRITARHIRQLEKDDVKLIEVPVEYIAGKVVAKD
YIDESTGELICAANMELSLDLLAKLSQSGHKRIETLFTNDLDHGPYISETLRVDPTNDRL
SALVEIYRMMRPGEPPTREAAESLFENLFFSEDRYDLSAVGRMKFNRSLLREEIEGSGIL
SKDDIIDVMKKLIDIRNGKGEVDDIDHLGNRRIRSVGEMAENQFRVGLVRVERAVKERLS
LGDLDTLMPQDMINAKPISAAVKEFFGSSQLSQFMDQNNPLSEITHKRRISALGPGGLTR
ERAGFEVRDVHPTHYGRVCPIETPEGPNIGLINSLSVYAQTNEYGFLETPYRKVTDGVVT
DEIHYLSAIEEGNYVIAQANSNLDEEGHFVEDLVTCRSKGESSLFSRDQVDYMDVSTQQV
VSVGASLIPFLEHDDANRALMGANMQRQAVPTLRADKPLVGTGMERAVAVDSGVTAVAKR
GGVVQYVDASRIVIKVNEDEMYPGEAGIDIYNLTKYTRSNQNTCINQMPCVSLGEPVERG
DVLADGPSTDLGELALGQNMRVAFMPWNGYNFEDSILVSERVVQEDRFTTIHIQELACVS
RDTKLGPEEITADIPNVGEAALSKLDESGIVYIGAEVTGGDILVGKVTPKGETQLTPEEK
LLRAIFGEKASDVKDSSLRVPNGVSGTVIDVQVFTRDGVEKDKRALEIEEMQLKQAKKDL
SEELQILEAGLFSRIRAVLVAGGVEAEKLDKLPRDRWLELGLTDEEKQNQLEQLAEQYDE
LKHEFEKKLEAKRRKITQGDDLAPGVLKIVKVYLAVKRRIQPGDKMAGRHGNKGVISKIN
PIEDMPYDENGTPVDIVLNPLGVPSRMNIGQILETHLGMAAKGIGDKINAMLKQQQEVAK
LREFIQRAYDLGADVRQKVDLSTFSDEEVMRLAENLRKGMPIATPVFDGAKEAEIKELLK
LGDLPTSGQIRLYDGRTGEQFERPVTVGYMYMLKLNHLVDDKMHARSTGSYSLVTQQPLG
GKAQFGGQRFGEMEVWALEAYGAAYTLQEMLTVKSDDVNGRTKMYKNIVDGNHQMEPGMP
ESFNVLLKEIRSLGINIELEDE
>sp|Q9L0L0|RPOB_STRCO DNA-directed RNA polymerase subunit beta
OS=Streptomyces coelicolor (strain ATCC BAA-471 / A3(2) / M145)
OX=100226 GN=rpoB PE=1 SV=1
MAASRNASTANTNNAASTAPLRISFAKIKEPLEVPNLLALQTESFDWLLGNDAWKARVES
ALESGQDVPTKSGLEEIFEEISPIEDFSGSMSLTFRDHRFEPPKNSIDECKDRDFTYAAP
LFVTAEFTNNETGEIKSQTVFMGDFPLMTNKGTFVINGTERVVVSQLVRSPGVYFDSSID
KTSDKDIFSAKIIPSRGAWLEMEIDKRDMVGVRIDRKRKQSVTVLLKALGWTTEQILEEF

GEYESMRATLEKDHTQGQDDALLDIYRKLRPGEPPTREAAQTLLENLYFNPKRYDLAKVG
RYKVNKKLGADEPLDAGVLTTDDVIATIKYLVKLHAGETETVGESGREIVVETDDIDHFG
NRRIRNVGELIQNQVRTGLARMERVVRERMTTQDVEAITPQTLINIRPVVASIKEFFGTS
QLSQFMDQNNPLSGLTHKRRLNALGPGGLSRERAGFEVRDVHPSHYGRMCPIETPEGPNI
GLIGSLASYGRINPFGFIETPYRKVVEGQVTDDVDYLTADEEDRFVIAQANAALGDDMRF
AEARVLVRRRGGEVDYVPGDDVDYMDVSPRQMVSVATAMIPFLEHDDANRALMGANMMRQ
AVPLIKSESPLVGTGMEYRSAADAGDVVKAEKAGVVQEVSADYITTTNDDGTYITYRLAK
FSRSNQGTSVNQKVIVAEGDRIIEGQVLADGPATENGEMALGKNLLVAFMPWEGHNYEDA
IILSQRLVQDDVLSSIHIEEHEVDARDTKLGPEEITRDIPNVSEEVLADLDERGIIRIGA
EVVAGDILVGKVTPKGETELTPEERLLRAIFGEKAREVRDTSLKVPHGEIGKVIGVRVFD
REEGDELPPGVNQLVRVYVAQKRKITDGDKLAGRHGNKGVISKINPIEDMPFLEDGTPVD
IILNPLAVPSRMNPGQVLEIHLGWLASRGWDVSGLAEEWAQRLQVIGADKVEPGTNVATP
VFDGAREDELAGLLQHTIPNRDGERMVLPSGKARLFDGRSGEPFPEPISVGYMYILKLHH
LVDDKLHARSTGPYSMITQQPLGGKAQFGGQRFGEMEVWALEAYGAAYALQELLTIKSDD
VTGRVKVYEAIVKGENIPEPGIPESFKVLIKEMQSLCLNVEVLSSDGMSIEMRDTDEDVF
RAAEELGIDLSRREPSSVEEV
>sp|P9WGY9|RPOB_MYCTU DNA-directed RNA polymerase subunit beta
OS=Mycobacterium tuberculosis (strain ATCC 25618 / H37Rv) OX=83332
GN=rpoB PE=1 SV=1
MLEGCILADSRQSKTAASPSPSRPQSSSNNSVPGAPNRVSFAKLREPLEVPGLLDVQTDS
FEWLIGSPRWRESAAERGDVNPVGGLEEVLYELSPIEDFSGSMSLSFSDPRFDDVKAPVD
ECKDKDMTYAAPLFVTAEFINNNTGEIKSQTVFMGDFPMMTEKGTFIINGTERVVVSQLV
RSPGVYFDETIDKSTDKTLHSVKVIPSRGAWLEFDVDKRDTVGVRIDRKRRQPVTVLLKA
LGWTSEQIVERFGFSEIMRSTLEKDNTVGTDEALLDIYRKLRPGEPPTKESAQTLLENLF
FKEKRYDLARVGRYKVNKKLGLHVGEPITSSTLTEEDVVATIEYLVRLHEGQTTMTVPGG
VEVPVETDDIDHFGNRRLRTVGELIQNQIRVGMSRMERVVRERMTTQDVEAITPQTLINI
RPVVAAIKEFFGTSQLSQFMDQNNPLSGLTHKRRLSALGPGGLSRERAGLEVRDVHPSHY
GRMCPIETPEGPNIGLIGSLSVYARVNPFGFIETPYRKVVDGVVSDEIVYLTADEEDRHV
VAQANSPIDADGRFVEPRVLVRRKAGEVEYVPSSEVDYMDVSPRQMVSVATAMIPFLEHD
DANRALMGANMQRQAVPLVRSEAPLVGTGMELRAAIDAGDVVVAEESGVIEEVSADYITV
MHDNGTRRTYRMRKFARSNHGTCANQCPIVDAGDRVEAGQVIADGPCTDDGEMALGKNLL
VAIMPWEGHNYEDAIILSNRLVEEDVLTSIHIEEHEIDARDTKLGAEEITRDIPNISDEV
LADLDERGIVRIGAEVRDGDILVGKVTPKGETELTPEERLLRAIFGEKAREVRDTSLKVP
HGESGKVIGIRVFSREDEDELPAGVNELVRVYVAQKRKISDGDKLAGRHGNKGVIGKILP
VEDMPFLADGTPVDIILNTHGVPRRMNIGQILETHLGWCAHSGWKVDAAKGVPDWAARLP
DELLEAQPNAIVSTPVFDGAQEAELQGLLSCTLPNRDGDVLVDADGKAMLFDGRSGEPFP
YPVTVGYMYIMKLHHLVDDKIHARSTGPYSMITQQPLGGKAQFGGQRFGEMECWAMQAYG
AAYTLQELLTIKSDDTVGRVKVYEAIVKGENIPEPGIPESFKVLLKELQSLCLNVEVLSS
DGAAIELREGEDEDLERAAANLGINLSRNESASVEDLA
>tr|X5EFQ6|X5EFQ6_NEIME DNA-directed RNA polymerase subunit beta
OS=Neisseria meningitidis OX=487 GN=rpoB PE=3 SV=1
MYMSYSFTEKKRIRKSFAKRENVLEVPFLLATQIDSYAKFLQLENAFDKRTDDGLQAAFN
SIFPIVSHNGYARLEFVHYTLGEPLFDIPECQLRGITYAAPLRARIRLVILDKEASKPTV
KEVRENEVYMGEIPLMTPSGSFVINGTERVIVSQLHRSPGVFFEHDKGKTHSSGKLLFSA
RIIPYRGSWLDFEFDPKDLLYFRIDRRRKMPVTILLKALGYNNEQILDIFYDKETFYLSS
NGVQTDLVAGRLKGETAKVDILDKEGNVLVAKGKRITAKNIRDITNAGLTRLDVEPESLL
GKALAADLIDSETGEVLASANDEITEELLAKFDINGVKEITTLYINELDQGAYISNTLRT
DETAGRQAARVAIYRMMRPGEPPTEEAVEQLFNRLFFSEDSYDLSRVGRMKFNTRTYEQK
LSEAQQNSWYGRLLNETFAGAADKGGYVLSVEDIVASIATLVELRNGHGEVDDIDHLGNR
RVRSVGELTENQFRSGLARVERAVKERLNQAESENLMPHDLINAKPVSAAIKEFFGSSQL

SQFMDQTNPLSEVTHKRRVSALGPGGLTRERAGFEVRDVHPTHYGRVCPIETPEGPNIGL
INSLSVYARTNDYGFLETPYRRVIDGKVTEEIDYLSAIEEGRYVIAQANADLDSDGNLIG
DLVTCREKGETIMATPDRVQYMDVATGQVVSVAASLIPFLEHDDANRALMGANMQRQAVP
CLRPEKPMVGTGIERSVAVDSATAIVARRGGVVEYVDANRVVVRVHDDEATAGEVGVDIY
NLVKFTRSNQSTNINQRPAVKAGDVLQRGDLVADGASTDLGELALGQNMTIAFMPWNGYN
YEDSILISEKVAADDRYTSIHIEELNVVARDTKLGAEDITRDIPNLSERMQNRLDESGIV
YIGAEVEAGDVLVGKVTPKGETQLTPEEKLLRAIFGEKASDVKDTSLRMPTGMSGTVIDV
QVFTREGIQRDKRAQSIIDSELKRYRLDLNDQLRIFDNDAFDRIERMIVGQKANGGPMKL
AKGSEITTEYLAGLPSRHDWFDIRLTDEDLAKQLELIKVSLQQKREEADELYEIKKKKLT
QGDELQPGVQKMVKVFIAIKRRLQAGDKMAGRHGNKGVVSRILPVEDMPYMADGRPVDIV
LNPLGVPSRMNIGQILEVHLGWAAKGIGERIDRMLKEQRKAGELREFLNRLYNGSGKKED
LDALTDEEIIELASNLRKGASFASPVFDGAKESEIREMLNLAYPSDDPEVEKLGFNDSKT
QITLYDGRSGEAFDRKVTVGVMHYLKLHHLVDEKMHARSTGPYSLVTQQPLGGKAQFGGQ
RFGEMEVWALEAYGAAYTLQEMLTVKSDDVNGRTKMYENIVKGEHKIDAGMPESFNVLVK
EIRSLGLDIDLERY
>sp|B8ZSC7|RPOB_MYCLB DNA-directed RNA polymerase subunit beta
OS=Mycobacterium leprae (strain Br4923) OX=561304 GN=rpoB PE=3 SV=1
MLEGCILPDFGQSKTDVSPSQSRPQSSPNNSVPGAPNRISFAKLREPLEVPGLLDVQTDS
FEWLIGSPCWRAAAASRGDLKPVGGLEEVLYELSPIEDFSGSMSLSFSDPRFDEVKAPVE
ECKDKDMTYAAPLFVTAEFINNNTGEIKSQTVFMGDFPMMTEKGTFIINGTERVVVSQLV
RSPGVYFDETIDKSTEKTLHSVKVIPSRGAWLEFDVDKRDTVGVRIDRKRRQPVTVLLKA
LGWTSEQITERFGFSEIMRSTLEKDNTVGTDEALLDIYRKLRPGEPPTKESAQTLLENLF
FKEKRYDLARVGRYKVNKKLGLHAGELITSSTLTEEDVVATIEYLVRLHEGQSTMTVPGG
VEVPVETDDIDHFGNRRLRTVGELIQNQIRVGMSRMERVVRERMTTQDVEAITPQTLINI
RPVVAAIKEFFGTSQLSQFMDQNNPLSGLTHKRRLSALGPGGLSRERAGLEVRDVHPSHY
GRMCPIETPEGPNIGLIGSLSVYARVNPFGFIETPYRKVVDGVVSDEIEYLTADEEDRHV
VAQANSPIDEAGRFLEPRVLVRRKAGEVEYVASSEVDYMDVSPRQMVSVATAMIPFLEHD
DANRALMGANMQRQAVPLVRSEAPLVGTGMELRAAIDAGHVVVAEKSGVIEEVSADYITV
MADDGTRRTYRMRKFARSNHGTCANQSPIVDAGDRVEAGQVIADGPCTENGEMALGKNLL
VAIMPWEGHNYEDAIILSNRLVEEDVLTSIHIEEHEIDARDTKLGAEEITRDIPNVSDEV
LADLDERGIVRIGAEVRDGDILVGKVTPKGETELTPEERLLRAIFGEKAREVRDTSLKVP
HGESGKVIGIRVFSHEDDDELPAGVNELVRVYVAQKRKISDGDKLAGRHGNKGVIGKILP
AEDMPFLPDGTPVDIILNTHGVPRRMNVGQILETHLGWVAKSGWKIDVAGGIPDWAVNLP
EELLHAAPNQIVSTPVFDGAKEEELQGLLSSTLPNRDGDVMVGGDGKAVLFDGRSGEPFP
YPVTVGYMYIMKLHHLVDDKIHARSTGPYSMITQQPLGGKAQFGGQRFGEMECWAMQAYG
AAYTLQELLTIKSDDTVGRVKVYEAIVKGENIPEPGIPESFKVLLKELQSLCLNVEVLSS
DGAAIELREGEDEDLERAAANLGINLSRNESASIEDLA
>sp|P60281|RPOB_MYCS2 DNA-directed RNA polymerase subunit beta
OS=Mycolicibacterium smegmatis (strain ATCC 700084 / mc(2)155)
OX=246196 GN=rpoB PE=1 SV=1
MLEGCILAVSSQSKSNAITNNSVPGAPNRVSFAKLREPLEVPGLLDVQTDSFEWLVGSDR
WRQAAIDRGEENPVGGLEEVLAELSPIEDFSGSMSLSFSDPRFDEVKASVDECKDKDMTY
AAPLFVTAEFINNNTGEIKSQTVFMGDFPMMTEKGTFIINGTERVVVSQLVRSPGVYFDE
TIDKSTEKTLHSVKVIPGRGAWLEFDVDKRDTVGVRIDRKRRQPVTVLLKALGWTNEQIV
ERFGFSEIMMGTLEKDTTSGTDEALLDIYRKLRPGEPPTKESAQTLLENLFFKEKRYDLA
RVGRYKVNKKLGLNAGKPITSSTLTEEDVVATIEYLVRLHEGQTSMTVPGGVEVPVEVDD
IDHFGNRRLRTVGELIQNQIRVGLSRMERVVRERMTTQDVEAITPQTLINIRPVVAAIKE
FFGTSQLSQFMDQNNPLSGLTHKRRLSALGPGGLSRERAGLEVRDVHPSHYGRMCPIETP
EGPNIGLIGSLSVYARVNPFGFIETPYRKVENGVVTDQIDYLTADEEDRHVVAQANSPTD
ENGRFTEDRVMVRKKGGEVEFVSADQVDYMDVSPRQMVSVATAMIPFLEHDDANRALMGA

NMQRQAVPLVRSEAPLVGTGMELRAAIDAGDVVVADKTGVIEEVSADYITVMADDGTRQS
YRLRKFARSNHGTCANQRPIVDAGQRVEAGQVIADGPCTQNGEMALGKNLLVAIMPWEGH
NYEDAIILSNRLVEEDVLTSIHIEEHEIDARDTKLGAEEITRDIPNVSDEVLADLDERGI
VRIGAEVRDGDILVGKVTPKGETELTPEERLLRAIFGEKAREVRDTSLKVPHGESGKVIG
IRVFSREDDDELPAGVNELVRVYVAQKRKISDGDKLAGRHGNKGVIGKILPVEDMPFLPD
GTPVDIILNTHGVPRRMNIGQILETHLGWVAKAGWNIDVAAGVPDWASKLPEELYSAPAD
STVATPVFDGAQEGELAGLLGSTLPNRDGEVMVDADGKSTLFDGRSGEPFPYPVTVGYMY
ILKLHHLVDDKIHARSTGPYSMITQQPLGGKAQFGGQRFGEMECWAMQAYGAAYTLQELL
TIKSDDTVGRVKVYEAIVKGENIPEPGIPESFKVLLKELQSLCLNVEVLSSDGAAIEMRD
GDDEDLERAAANLGINLSRNESASVEDLA
>sp|A1KGE7|RPOB_MYCBP DNA-directed RNA polymerase subunit beta
OS=Mycobacterium bovis (strain BCG / Pasteur 1173P2) OX=410289 GN=rpoB
PE=3 SV=2
MLEGCILADSRQSKTAASPSPSRPQSSSNNSVPGAPNRVSFAKLREPLEVPGLLDVQTDS
FEWLIGSPRWRESAAERGDVNPVGGLEEVLYELSPIEDFSGSMSLSFSDPRFDDVKAPVD
ECKDKDMTYAAPLFVTAEFINNNTGEIKSQTVFMGDFPMMTEKGTFIINGTERVVVSQLV
RSPGVYFDETIDKSTDKTLHSVKVIPSRGAWLEFDVDKRDTVGVRIDRKRRQPVTVLLKA
LGWTSEQIVERFGFSEIMRSTLEKDNTVGTDEALLDIYRKLRPGEPPTKESAQTLLENLF
FKEKRYDLARVGRYKVNKKLGLHVGEPITSSTLTEEDVVATIEYLVRLHEGQTTMTVPGG
VEVPVETDDIDHFGNRRLRTVGELIQNQIRVGMSRMERVVRERMTTQDVEAITPQTLINI
RPVVAAIKEFFGTSQLSQFMDQNNPLSGLTHKRRLSALGPGGLSRERAGLEVRDVHPSHY
GRMCPIETPEGPNIGLIGSLSVYARVNPFGFIETPYRKVVDGVVSDEIVYLTADEEDRHV
VAQANSPIDADGRFVEPRVLVRRKAGEVEYVPSSEVDYMDVSPRQMVSVATAMIPFLEHD
DANRALMGANMQRQAVPLVRSEAPLVGTGMELRAAIDAGDVVVAEESGVIEEVSADYITV
MHDNGTRRTYRMRKFARSNHGTCANQCPIVDAGDRVEAGQVIADGPCTDDGEMALGKNLL
VAIMPWEGHNYEDAIILSNRLVEEDVLTSIHIEEHEIDARDTKLGAEEITRDIPNISDEV
LADLDERGIVRIGAEVRDGDILVGKVTPKGETELTPEERLLRAIFGEKAREVRDTSLKVP
HGESGKVIGIRVFSREDEDELPAGVNELVRVYVAQKRKISDGDKLAGRHGNKGVIGKILP
VEDMPFLADGTPVDIILNTHGVPRRMNIGQILETHLGWCAHSGWKVDAAKGVPDWAARLP
DELLEAQPNAIVSTPVFDGAQEAELQGLLSCTLPNRDGDVLVDADGKAMLFDGRSGEPFP
YPVTVGYMYIMKLHHLVDDKIHARSTGPYSMITQQPLGGKAQFGGQRFGEMECWAMQAYG
AAYTLQELLTIKSDDTVGRVKVYEAIVKGENIPEPGIPESFKVLLKELQSLCLNVEVLSS
DGAAIELREGEDEDLERAAANLGINLSRNESASVEDLA
>tr|S5G1A7|S5G1A7_9MYCO DNA-directed RNA polymerase subunit beta
(Fragment) OS=Mycobacteroides abscessus subsp. bolletii OX=319705
GN=rpoB PE=3 SV=1
VQTESFEWLVGSPRWREVATARGEVNPTGGLEEILTELSPIEDFSGSMSLSFSDPRFDEV
KAPVDECKDKDMTYAAPLFVTAEFINNNTGEIKSQTVFMGDFPMMTDMGTFIINGTERVV
VSQLVRSPGVYFDESIDKSTEKTLHSVKVIPGRGAWLEFDVDKRDTVGVRIDRKRRQPVT
VLLKALGWTNEQIVERFGFSEIMMGTLEKDNIAGPDEALLDIYRKLRPGEPPTKESAQAL
LENLFFKEKRYDLARVGRYKVNKKLGLGGTNPAQVTTTTLTEEDVVATIEYLVRLHEGQT
TMTAPGGVEVPVDVDDIDHFGNRRLRTVGELIQNQIRVGLSRMERVVRERMTTQDVEAIT
PQTLINIRPVVAAIKEFFGTSQLSQFMDQNNPLSGLTHKRRLSALGPGGLTRDRAGLEVR
DVHPSHYGRMCPIETPEGPNIGLIGSLSVYARVNPFGFIETPYRKVSDGVVTDEIHYLTA
DEEDRHVVAQANSPVDANGRFTEEKILVRRKGGEVEFVSATEVDYMDVSPRQMVSVATAM
IPFLEHDDANRALMGANMQRQAVPLVRSEAPLVGTGMELRAAIDAGDVVVAEKAGVIEEV
SADYVTVMADDGTRQSYRLRKFARSNHGTCANQKPIVDEGQRVEAGQVVADGPCTENGEM
ALGKNLLVAVMPWEGHNYEDAIILSNRLVEEDVLTSIHIEEHEIDARDTKLGAEEITRDI
PNVSDEVLADLDERGIVRIGAEVRDGDILVGKVTPKGETELTPEERLLRAIFGEKAREVR
DTSLKVPHGESGKVIGIRVFSRDDDDDLPAGVNELVRVYVAQKRKISDGDKLAGRHGNKG

VIGKILPVEDMPFLPDGTPVDIILNTHGVPRRMNIGQILETHLGWIAKTGWNIEGDPEWA
QNLPEDLQSAPADTRTATPVFDGAREEELTGLLSSTLPNRDGEVMVDGDGKARLFDGRSG
EPFPYPVTVGYMYILKLHHLVDDKIHARSTGPYSMITQQPLGGKAQFGGQRFGEMECWAM
QAYGAAYTLQELLTIKSDDTVGRVKVYEAIVKGENIPEPGIPESFKVLLKELQSLCLNVE
VLSKDGAAIEMRDGDDEDLERAAANLGINLSRNESASIEDFA
>sp|Q9L637|RPOB_AMYMS DNA-directed RNA polymerase subunit beta
OS=Amycolatopsis mediterranei (Nocardia) (strain S699) OX=713604
GN=rpoB PE=3 SV=2
MAVSPANQATAATTSAESRSEATGIPGAPKRVSFAKIREPLNTPNLLDVQIQSFQWFTGD
EAWFQRRVEEGEENPVGGLEEVLNEISPIEDFSGSMSLSFSAPRFDEVKASIEECKDKDM
TYAAPLFVTAEFVNNNTGEIKSQTVFLGDFPVMTDKGTFVINGTERVVVSQLVRSPGVYY
SKDIDKTSDKDVFSVRVIPSRGAWLEFDVDKRDTVGVRIDRKRRQPVTVLLKALGWTTEA
IRERFSFSETLLATLEKDHTAGTDEALLDIYRKLRPGEPPTKESAQTLLENLFFKAKRYD
LAKVGRYKVNKKLGLSTPIENGTLTEEDIVTIIEYLVRLHAGEDKMTAANNTEIPVETDD
IDHFGNRRIRTVGELIQNQIRVGLSRTERVVRERMTTQDVEAITPQTLINIRPIGAAIKE
FFGTSQLSQFMQQTNPIDGLTHKRRLNALGPGGLSRERAGMEVRDVHPSHYGRMCPIETP
EGPNIGLIGSLCSYARVNPFGFIETPYRKVVEGRVTDQIDYLTADEEDRFVKAQANAPIS
DDGTFIEDRVMARRKGGEVELIDPLDIDYMDVSPRQMVSIATAMIPFLEHDDANRALMGA
NMQRQAVPLLRSQAPYVGTGVELRAAIDSGDMLVAEQSGVVEELSADLITVMHDDGTRKS
YSLYKFRRSNHGTCFNHRPIVNEGDRIEAGQVIADGPSTENGEVALGKNLLVAVMPWEGH
NYEDAIILSERLVQDDVLTSIHIEEHEIDARDTKLGAEEITRDIPNVSEEVLADLDERGI
IRIGAEVRDGDILVGKVTPKGETELTPEERLLRAIFGEKAREVRDTSLKVPHGETGKVIG
IRVFSREDDDELPPGVNELVRVYVAQKRKIQPGDKLAGRHGNKGVIGKILPVEDMPFMED
GTPVDIILNTHGVPRRMNIGQILELHLGWLASQGWTIEGDPDWAKNLSAELRDVAPGTNT
ATPVFDGAKEEELTGLLSATKPNRDGERMVKENGKANLFDGRSGEPYPYPVAVGYMYILK
LHHLVDDKIHARSTGPYSMITQQPLGGKAQFGGQRFGEMECWAMQAYGAAYTLQELLTIK
SDDVVGRVKVYEAIVKGENIPEPGIPESFKVLLKELQSLCLNVEVLSSDGSSIEMRDSDD
EDLERAAANLGINLSRNESPSVDDVVH
>tr|A0A1J5VR89|A0A1J5VR89_9CORY DNA-directed RNA polymerase subunit
beta OS=Corynebacterium sp. NML130628 OX=1906333 GN=rpoB PE=3 SV=1
MLEGPNLAVSNQTMSMAEIPGAPERYSFAKIAEPIAVPGLLDVQLESFAWLVGTQEWRER
EQEARGADARVTSGLEDILEEISPIQDYSGNMSLTLSEPRFEDVKDTIDECKEKDINYSA
PLYVTAEFINNETQEIKSQTVFIGDFPLMTDKGTFIVNGTERVVVSQLVRSPGVYFDETI
DKSTERPLHAVKVIPSRGAWLEFDVDKRDTVGVRIDRKRRQPVTVLLKALGWTTEQITER
FGFSEIMMSTLENDGVANTDEALLEIYRKQRPGEQPTRDLAQSLLENSFFKAKRYDLARV
GRYKVNRKLGLGGDHDGLMTLTEEDIATTLEYLVRLHAGETEMTSPEGEVIAINTDDIDH
FGNRRLRTVGELIQNQVRVGLSRMERVVRERMTTQDAESITPTSLINVRPVSAAIREFFG
TSQLSQFMDQNNSLSGLTHKRRLSALGPGGLSRERAGIEVRDVHPSHYGRMCPIETPEGP
NIGLIGALSSYARVNAFGFIETPYQKVEDGKLTDTVHYLTADEEDRYAIAQAATPMDKDR
NLTGERIEVRLKDGDIGVVGPKGVDYLDISPRQMVSVATAMIPFLEHDDANRALMGANMQ
KQAVPLLRSEAAYVATGMEQRAAYDAGDTIISAKAGVVTNVTGDFITVMDDEGIQDTYML
RTFERTNQGTCYNQVPIVDHGQRVEAGQVLADGPGTKNGEMALGRNLLVAFMPWEGHNYE
DAIILNQRVVEDDILTSVHIEEHEIDARDTKLGAEEITREIPNVSEDVLKDLDERGIIRI
GADVRDGDILVGKVTPKGETELTPEERLLRAIFGEKAREVRDTSLKVPHGETGKVIAVRR
FSREDDDDLSPGVNEMIRVYVAQKRKIQDGDKMAGRHGNKGVVGKILPQEDMPFMADGTP
VDIILNTHGVPRRMNIGQVLEVHLGWLAKAGWTVNPDDPANAKLLETLPEHLYDVPPESL
TATPVFDGATNEEIAGLLANSKPNRDGDVMVDENGKTTLFDGRSGEPFKYPISVGYMYML
KLHHLVDEKIHARSTGPYSMITQQPLGGKAQFGGQRFGEMEVWAMQAYGAAYTLQELLTI
KSDDVVGRVKVYEAIVKGENIPDPGIPESFKVLLKELQSLCLNVEVLSTDGTPMELDGDD
DDFDQAGSSLGINLSRDEGSATDTA

>tr|F2RIS5|F2RIS5_STRVP DNA-directed RNA polymerase subunit beta
OS=Streptomyces venezuelae (strain ATCC 10712 / CBS 650.69 / DSM
40230 / JCM 4526 / NBRC 13096 / PD 04745) OX=953739 GN=rpoB PE=1 SV=1
MQGTGTRVVSPSPRKDPLLAASRNASTNTNNGASTAPLRISFAKIKEPLEVPNLLALQTE
SFDWLLGNAAWKARVEAALESGQDVPTKSGLEEIFEEISPIEDFSGSMSLTFRDHRFEPP
KNSIDECKDRDFTYGAPLFVTAEFTNNETGEIKSQTVFMGDFPLMTNKGTFVINGTERVV
VSQLVRSPGVYFDSSIDKTSDKDIFSAKIIPSRGAWLEMEIDKRDLVGVRIDRKRKQSVT
VLLKALGWTTEQILQEFGEYESMRATLEKDHTQGQDDALLDIYRKLRPGEPPTREAAQTL
LENLYFNPKRYDLAKVGRYKVNKKLGADEPLDAGVLTTDDVIATIKYLVKLHAGETETIG
ENGNEIVVETDDIDHFGNRRLRNVGELIQNQVRTGLARMERVVRERMTTQDVEAITPQTL
INIRPVVASIKEFFGTSQLSQFMDQNNPLSGLTHKRRLSALGPGGLSRERAGFEVRDVHP
SHYGRMCPIETPEGPNIGLIGSLASYGRVNAFGFIETPYRKVVDGQVTDEVDYVTADEED
RFVIAQANAALDEELRFSENRVLVRKRGGEVDYVEPSDVDYMDVSPRQMVSVATAMIPFL
EHDDANRALMGANMMRQAVPLIKSEAPLVGTGMEYRCATDAGDVLKAEKDGVVQELSADY
ITVANDDGTYITYRLHKFSRSNQGTSVNQKVVVDEGDRVIEGQVLADGPATEDGEMALGK
NLLVAFMPWEGHNYEDAIILSQRLVQDDVLSSIHIEEHEVDARDTKLGPEEITRDIPNVS
EEVLADLDERGIIRIGAEVVAGDILVGKVTPKGETELTPEERLLRAIFGEKAREVRDTSL
KVPHGEIGKIIGVRVFDREEGDELPPGVNQLVRVYVAQKRKITDGDKLAGRHGNKGVISK
ILPIEDMPFLEDGTPVDIILNPLGVPSRMNPGQVLEIHLGWLASRGWDVSGLADEWAQRL
QAIGADKVAPGTNVATPVFDGAREDELAGLLNHTIPNRDGERMVLPTGKARLFDGRSGEP
FPDPISVGYMYILKLHHLVDDKLHARSTGPYSMITQQPLGGKAQFGGQRFGEMEVWALEA
YGAAYALQELLTIKSDDVTGRVKVYEAIVKGENIPEPGIPESFKVLIKEMQSLCLNVEVL
SSDGMSIEMRDTDEDVFRAAEELGIDLSRREPSSVEEV
>tr|A0A380PA28|A0A380PA28_STRGR DNA-directed RNA polymerase subunit
beta OS=Streptomyces griseus OX=1911 GN=rpoB PE=3 SV=1
MAASRTASANSNNGASTAPLRISFAKIKEPLEVPNLLALQTESFDWLLGNDAWKARVEAA
LDSGQDVPTKSGLEEIFEEISPIEDFSGSMSLTFRDHRFEPPKNSIDECKERDFTFAAPL
FVTAEFTNNETGEIKSQTVFMGDFPLMTNKGTFVINGTERVVVSQLVRSPGVYFDSSIDK
TSDKDIFSAKIIPSRGAWLEMEIDKRDMVGVRIDRKRKQSVTVLLKALGWTTEQILEEFG
EYESMRATLEKDHTQGQDDALLDIYRKLRPGEPPTREAAQTLLENLYFNPKRYDLAKVGR
YKVNKKLGGDAPLDAGVLTTDDVIATIKYLVKLHAGETETTGENGTQIVVETDDIDHFGN
RRLRNVGELIQNQVRTGLARMERVVRERMTTQDVEAITPQTLINIRPVVASIKEFFGTSQ
LSQFMDQNNPLSGLTHKRRLSALGPGGLSRERAGFEVRDVHPSHYGRMCPIETPEGPNIG
LIGSLASYGRVNAFGFVETPYRRVTEGVVTDEVDYLTADEEDRFVIAQANAGLTDDLHFA
EDRVLVRRRGGEVDYVPGDDVDYMDVSPRQMVSVATAMIPFLEHDDANRALMGANMMRQA
VPLIKSEAPLVGTGMEYRCATDAGDVLKAEKDGVVQEVSADYITTTNDDGTYTTYRLAKF
SRSNQGTSVNQKVVVDEGDRIVEGQVLADGPATEQGEMALGKNLLVAFMSWEGHNYEDAI
ILSQRLVQDDVLSSIHIEEHEVDARDTKLGPEEITRDIPNVSEEVLADLDERGIIRIGAE
VDAGDILVGKVTPKGETELTPEERLLRAIFGEKAREVRDTSLKVPHGETGKVIGVRVFDR
EEGDELPPGVNQLVRVYVAQKRKITDGDKLAGRHGNKGVISKILPIEDMPFLEDGTPVDI
ILNPLGVPSRMNPGQVMEIHLGWLASQGWDISGVDTEWAERLQAIGIDRVEPGTNVATPV
FDGTREDELAGLIDHTIPNRDGERMVQSSGKARLFDGRSGEPFPDPISVGYMYILKLHHL
VDDKLHARSTGPYSMITQQPLGGKAQFGGQRFGEMEVWALEAYGAAYALQELLTIKSDDV
TGRVKVYEAIVKGENIPEPGIPESFKVLIKEMQSLCLNVEVLSSDGMSIEMRDTDEDVFR
AAEELGIDLSRREPSSVEEV
>sp|B3DTE2|RPOB_BIFLD DNA-directed RNA polymerase subunit beta
OS=Bifidobacterium longum (strain DJO10A) OX=205913 GN=rpoB PE=3 SV=1
MATESTTNTTTIIARADQHDIDLHKASDRVNFGSIKEPIDVPYLLGVQTDSFDWLIGNER
WKARVEEDEKNGTNTVAHTSGLDEVFNEISPIENFAQTMSLTFSDPYFEEPRHTVQECKE
KDYTYSAPLYVNAEFENGDTGEIKSQTVFMGDFPLQTPHGTFIIGGTERVIVSQLVRSPG

VYFDRQQDRTSDKEVFGAKIIPSRGAWLEFEIDKKDQPQVRVDRKRKQSAIVFLMAIGMT
KSEIAQAFKDYPLVLDALEKETLGTQDEALVDLYRKIRPADTPTPEAGKNLLDSFYFNTK
RYDLARVGRYKINRKLGVEADFNDRSLHQEDIIATIKYLVALHDGAATFPGKRNGEDVDL
RVDVDDIDHFGNRRIRQVGELIQNQLRTGLSRMERVVRERMTTQDAEAITPQSLINIRPV
NATIKEFFGTSQLSQFMDQNNPLSGVTNKRRLSALGPGGLSRDRASMEVRDVHPSHFGRM
CPIESPEGPNIGLIGSLATFGRVNPFGFIETPYRKVVNGHVTDEVEYMTADRDLDHVIAQ
ANQELDENGNFVQKSALARVGEEEAVDVPVSSVDYMDVSPRQMVSLGASLIPFLEHDEGH
RALMGTNMQRQAVPLIESERPLVGTGSEWRAANDSGDVIKSEKDGVVTYVSADLIRVMND
DGTTSSYKLAKFQRSNQTTCYNQRPIVHDGERVEAGSVMADGPAIQNGDLALGKNLLIAF
MPWNGYNYEDAVIISQRLVQDDTLSSIHIEEYEIDARETKLGAEEITRDLPNVGEDAVAN
LDERGIIRIGAEVEAGDILVGKVTPKGETELTPEERLLRAIFGEKSREVRDTSLRVPHGE
TGTVIGVKEITREDAEEDGDELPNGVNQMIRVYIAQHRKITVGDKLSGRHGNKGCISRIL
PEEDMPFLADGTPVDIMLNPLGVPSRMNLGQVLELHLGWIAHSGWDISLDPNLEAEWKKL
IPSGAEKAEPNTPVATPVFDGVKPEVLKGLLSTTLPNRDGDRLVGPDGKATLFDGRTGEP
YTKPISVGYMYMLKLHHLVDDKIHARSTGPYSMITQQPLGGKAQFGGQRFGEMEVWALEA
YGAAYTLHEMMTTKSDDVDGRVRVYGAIVKGDNLPPAGIPESFKVLLKEMQSLSLNVEVL
NAEGVAIDMKDEDDDPASSADDLGFNIGARPDAAAKEDQKAEEPEYQ
>tr|A0A378NJ65|A0A378NJ65_MICLU DNA-directed RNA polymerase subunit
beta OS=Micrococcus luteus OX=1270 GN=rpoB PE=3 SV=1
MVASSTSNETATVSPRSGASAGRVSFAKISEPLDVPDLLALQTESFDRLIGNERWAARVD
AALAADDHSVPVTSGLTDIFEEISPIEDFQGTMSLSFAEPEFADAKMTEEECKDRDATFS
APLYVKAEFMNNTTGEIKQQTVFMGDFPLMTRNGTFIINGTERVVVSQLVRSPGAYFERT
PDKTSDKEIFSAKIIPSRGAWFELEVDKRDQVGVRLDRKRKQPVTVLLKAMGWTESRILE
EFGHYDSIRATLEKDGTADQDEALLDIYRKLRPGEPAAVDAAQTLLNNLYFTPKRYDLAK
VGRYKLNRKLGVDVHLGDPNASVLTEDDIVQMVHFIAALHAGETAIKGTRDGEPVDVRVE
VDDIDHFGNRRIRAVGELIENQIRTGLSRMERVVRERMTTQDVEAITPQTLINIRPVVAA
IKEFFGTSQLSQFMDQNNPLAGLTHKRRLSALGPGGLSRDRAGMEVRDVHPSHYGRMCPI
ETPEGPNIGLIGSLATFGRINSFGFIETPYRRVVDGRVTDTVDYLTADDELAFQIAQANA
PVDEDGRFEEELVLCRQRGGDGEPVLSTVDDIDYMDVSPRQMVSAATALIPFLEHDDANR
ALMGANMQRQAVPLLRSEAPYVGTGMEKYVAVDAGDSVTATQGGVVTEVAADLVTVMNDD
GTTTHYPIMKFARSNQGNAYNQRVRVSEGERVEPLSIIADGPATDNGELALGKNLLVAFM
PWEGLNYEDAIILSQRMVSEDVLTSIHIEEHEVDARDTKLGAEEITRDIPNVSEEVLSQL
DERGIIHIGAEVEAGDILVGRVTPKGETELTPEERLLRAIFGEKSREVRDTSLKVPHGES
GTVIGVRIFDRDEDDDLPPGVNQLVRVYVAQKRKITDGDKMAGRHGNKGVISKILPLEDM
PFLADGTPVDIVLNPLGVPGRMNLGQVMELHLGWAASRGWDIQGEPEWIKDLPNFPRQSG
PVNVATPVFDGAEAYEITGLLGHVNVTRDGDRLMSDSGKAQLFDGRTGEPFPDPVSVGYM
YMLKLHHLVDDKIHARSTGPYSMITQQPLGGKAQFGGQRFGEMEVWALEAYGAAFTLQEL
LTIKSDDIHGRVKVYEAIVKGENIPEPGVPESFKVLIKEMQSLCLNVEVLSADGQAIEMR
DAEDEVYRAAEELGIDLSHAEPSSVEEV
>sp|Q47LI5|RPOB_THEFY DNA-directed RNA polymerase subunit beta
OS=Thermobifida fusca (strain YX) OX=269800 GN=rpoB PE=3 SV=1
MAASRIASANALGPNRVSFARIKEPLEVPNLLALQTESFDWLLGNERWRARVEAARKAGR
KDIPEQSGLEEIFEEISPIEDFSGTMSLSFRDHRFEPPKYSEEECKDKDMTYSAPMFVTA
EFINNDTGEIKSQTVFMGDFPLMTAKGTFIINGTERVVVSQLVRSPGVYFDSQMDKSSDK
ELYGCKIIPSRGAWLEFEIDKRDFVGVRIDRKRKQAVTILLKALGWTTDQILERFGEYES
IRATLEKDPTAGTDDALLDIYRKLRPGEPPTKEAAQALLENLYFNPKRYDLAKVGRYKIN
KKLGLEIDITQGTLTEEDIVATVDYLVRLHAGEKELVRPHGTFPIEVDDIDHFGNRRLRT
VGELIQNQVRLGLARMERVVRERMTTQDVEAITPQTLINIRPVVASIREFFGTSQLSQFM
DQTNPLAGLTHKRRLSALGPGGLSRERAGFEVRDVHPSHYGRMCPIETPEGPNIGLIGSL
AAYARVNSFGFIETPYRKVVDGRITDEVVYLTADEEDRYVIAQANTPVNPDGTFAESQVL

ARRKGGEFESVAAEEVHYMDISPRQMVSVATAMIPFLEHDDANRALMGSNMQRQAVPLLR
AEAPLVGTGMEYRAATDAGDVILAEKSGVVEDVTADYITVLADDGTRKTYRVHKFRRTNQ
GTCFNQRPIVEEGQRVEEGQVLADGPSTEAGEMALGKNLLVAYMSWEGHNYEDAIVLSQR
LVEEDILSSIHIEEHEVDARETKLGPEEITREIPNVSEEVLADLDERGIIRIGAEVVDGD
ILVGKVTPKGETELTPEERLLRAIFGEKAREVRDTSLKVPHGESGKVIGVRVFSREEGDE
LPPGVNELVRVYVAQKRKITDGDKLAGRHGNKGVIAKILPKEDMPFLEDGTPVDIVLNPL
GVPGRMNIGQIMEMHLGWLAKHGWKVEGDDAEWKRRLRDIGAHEAPPNSKVATPVFDGAR
EDEISGLLSCVLPDQDGDILVNKFGKAKLYDGRTGEPFKEPVAVGYAYFLKLHHLVDDKI
HARSTGPYSMITQQPLGGKAQFGGQRFGEMEVWALEAYGAAYALQELLTIKSDDINGRVK
VYEAIVKGENIPEPGIPESFKVLIKEMQSLCLNVEVLSRDGMSIEMRDSEEDVFRAAEEL
GIDLGRREPSSVEEV
>sp|Q6A6K6|RPOB_CUTAK DNA-directed RNA polymerase subunit beta
OS=Cutibacterium acnes (Propionibacterium) (strain DSM 16379 /
KPA171202) OX=267747 GN=rpoB PE=3 SV=1
MAATRTASKNTSAISPQSGRISFAKIHEPLEVPNLLDLQVESFNWLVGNEIWQSRVDAAL
AEGRTDINTKSGLEEIFEEISPIEDYSQTMSLSFRDHRFEDPKHSVDECKDRDTTYAAPL
FVTAEFMNNETEEIKSQTVFIGDFPLMTSKGTFIINGTERVVVSQLVRSPGVYFEKTADK
TSDKDIFTCKVIPSRGAWLEFAIDKRDTVGVRLDRKRKQNVTVFLKALGWTADRILEEFG
THESIRQTLEKDHGVETQDQALLDIYKKLRPGEPPSRDAAQQLLENYYFNPKRYDLAKVG
RYKINKKLGLSLPFDQQVLTVDDIVAAIHFVCALHEGTAILPREGQDDIVVEPDDIDHFG
NRRLRTVGELIQNQLRTGLSRMERVVRDRMTTQDIEAITPQTLINIRPVTAAIKEFFGTS
QLSQFMDQNNPLAEMTHKRRLSALGPGGLSRDRAGMEVRDVHPSHYGRMCPIETPEGPNI
GLIGSLASFARVNAFGFIETPYRKVVDGHVTDEVVYLTADEEDRHVIAQANAKLDDDGHF
ANDRVLVRQRHGEADEVPSSEVDYMDVSPRQMVSVASALIPFLEHDDASRALMGANMQRQ
AVPLVRTEAPFVGTGMEYRCAVDVGDVTLAEKAGSVLSVSADLIDIACDDGTYQTYKLEK
FRRSNAGTCINQRPLVKVGQRVEVGTPLADGPSTDNGELALGRNMLAAFMPWQGLNYEDA
IILSQRIVSDDVLTSIHIEEHEVDARDTKLGAEEITRDIPNVSEDMLANLDENGIVRIGA
EVGTGDILVGKVTPKGETELTPEERLLRAIFGEKAREVRDTSLKVPHGEEGTVIGVRIFD
TENGDELAPGVNQMVRVYVAQKRKISIGDKLAGRHGNKGVISKILPVEDMPFLPDGTPVD
IILNPLGVPSRMNVGQVLEMHLGWIAHSGWDITQAEGDWAERLREVGLIDIPEESRLATP
VFDGATEQEITGLLQYGHPTRDGEMLVDTDGKATLFDGRTGEPFPSKVGVGYMYMLKLHH
LVDDKIHARSTGPYSMITQQPLGGKAQFGGQRFGEMEVWAMEAYGAAWALQELLTIKSDD
VPGRVKVYEAIVKGENIPEPGIPESFKVLVKEMKSLCLNVEVLNSEGQEIDLRGSEEDTR
SGLGIDIGRRPGADNAMAD
>sp|Q2JFI5|RPOB_FRACC DNA-directed RNA polymerase subunit beta
OS=Frankia casuarinae (strain DSM 45818 / CECT 9043 / HFP020203 /
CcI3) OX=106370 GN=rpoB PE=3 SV=1
MAASRSSSRISFAKIIEPLEVPDLLALQTQSFDWLIGSDAWAERVQEAIDSGRDDVPITS
GLEEVFKEISPIEDFSGSMSLSFRDHRFEPPKYSVEECKDKDMTFSAPLFVTAEFTNNNT
GEIKSQTVFMGDFPLMTPKGTFVINGTERVVVSQLVRSPGVYFERSLDKASDKDLYSCKV
IPSRGAWLEFEIDKRDTVGVRIDRKRRQSVTVLLKALGWDEARILERFGDFPSMRITLEK
DHTAGQDDALLDIYRKLRPGEPPTRESAQTLLENLFFNPKRYDLAKVGRYKVNKKLSLGV
AHDVGVLTENDIVRTIEYVVKLHAGADPAEYEVDDIDHFGNRRIRTVGELIQNQVRLGLA
RMERVVRERMTTQDVEAITPQTLINIRPVVASIKEFFGTSQLSQFMDQTNPLAGLTHKRR
LNALGPGGLSRERAGFEVRDVHPSHYGRMCPIETPEGPNIGLIGSLSTFARVNPFGFIET
PYRKVENGRVTGQIDWLTADEEDRHVKAQANTPLRPDGSFAEDRVLVRRKGGEVEFIPPD
EVDYMDVSPRQMVSVATAMIPFLEHDDANRALMGSNMQRQSVPLLRSEAPLVGTGMEARA
AKDAGDVVVCAQAGVVEDLSADYITVMHDDGTRRTYRLAKFRRSNQGTCINQKPIVNEGD
RVGAGQVIADGPCTDNGEMALGKNLLVAFMPWEGHNYEDAIILSQRLVQDDVLSSIHIEE
HEVDARDTKLGPEEITRDIPNVAEEVLADLDERGIIRIGAEVSPGDVLVGKVTPKGETEL

TPEERLLRAIFGEKAREVRDTSLKVPHGESGKVIGVRVFSREDGDELPPGVNELVRVYVA
QKRKITDGDKLAGRHGNKGVIAKILPAEDMPFLEDGTPVDVVLNPHGVPRRMNIGQILET
HLGWVAKTGWQVDSGTEDWKERLRGIGADAASAGTNVATPVFDGAREEEITGLLDSTLPN
RDGIQLIGSSGKAKLFDGRTGEPYPYPVAVGYIYILKLLHLVDDKIHARSTGPYSMITQQ
PLGGKAQFGGQRFGEMEVWALEAYGAAYALQELLTIKSDDVVGRVKVYEAIVKGENIPEP
GIPESFKVLIKEMQSLCLNVEVLSSDGVQIEMRDTDEDVFRAAEELGIDLSRREPSSVEE
V
>sp|A0QL49|RPOB_MYCA1 DNA-directed RNA polymerase subunit beta
OS=Mycobacterium avium (strain 104) OX=243243 GN=rpoB PE=3 SV=2
MLEGCILADFRQSKTDRPQSSSNGSSSLNGSVPGAPNRVSFAKLREPLEVPGLLDVQIDS
FEWLIGAPRWREAAIARGDAEPKGGLEEVLDELSPIEDFSGSMSLSFSDPRFDEVKAPVD
ECKDKDMTYAAPLFVTAEFINNNTGEIKSQTVFMGDFPMMTEKGTFIINGTERVVVSQLV
RSPGVYFDETIDKSTEKTLHSVKVIPSRGAWLEFDVDKRDTVGVRIDRKRRQPVTVLLKA
LGWTNEQITERFGFSEIMMSTLEKDNTAGTDEALLDIYRKLRPGEPPTKESAQTLLENLF
FKEKRYDLARVGRYKVNKKLGLHAGEPITSSTLTEEDVVATIEYLVRLHEGQPTMTVPGG
IEVPVETDDIDHFGNRRLRTVGELIQNQIRVGMSRMERVVRERMTTQDVEAITPQTLINI
RPVVAAIKEFFGTSQLSQFMDQNNPLSGLTHKRRLSALGPGGLSRERAGLEVRDVHPSHY
GRMCPIETPEGPNIGLIGSLSVYARVNPFGFIETPYRKVVDGVVTDEIHYLTADEEDRHV
VAQANSPIDDKGRFAEARVLVRRKAGEVEYVPSSEVDYMDVSPRQMVSVATAMIPFLEHD
DANRALMGANMQRQAVPLVRSEAPLVGTGMELRAAIDAGDVVVAEKSGVIEEVSADYITV
MADDGTRHTYRMRKFERSNHGTCANQSPIVDAGDRVEAGQVIADGPCTENGEMALGKNLL
VAIMPWEGHNYEDAIILSNRLVEEDVLTSIHIEEHEIDARDTKLGAEEITRDIPNVSDEV
LADLDERGIVRIGAEVRDGDILVGKVTPKGETELTPEERLLRAIFGEKAREVRDTSLKVP
HGESGKVIGIRVFSREDDDELPAGVNELVRVYVAQKRKISDGDKLAGRHGNKGVIGKILP
QEDMPFLPDGTPVDIILNTHGVPRRMNIGQILETHLGWVAKSGWNIDGNPEWAVNLPEEL
RHAQPNQIVSTPVFDGAKEEELAGMLSCTLPNRDGEVMVDGDGKAVLFDGRSGEPFPYPV
TVGYMYIMKLHHLVDDKIHARSTGPYSMITQQPLGGKAQFGGQRFGEMECWAMQAYGAAY
TLQELLTIKSDDTVGRVKVYEAIVKGENIPEPGIPESFKVLLKELQSLCLNVEVLSSDGA
AIELREGEDEDLERAAANLGINLSRNESASVEDLA
